# Pure flavonoid epicatechin and whole genome gene expression profiles in circulating immune cells in adults with elevated blood pressure: A randomised double-blind, placebo-controlled, crossover trial

**DOI:** 10.1371/journal.pone.0194229

**Published:** 2018-04-19

**Authors:** Diederik Esser, Johanna M. Geleijnse, Juri C. Matualatupauw, James I. Dower, Daan Kromhout, Peter C. H. Hollman, Lydia A. Afman

**Affiliations:** 1 Top Institute Food and Nutrition, Wageningen, The Netherlands; 2 The Division of Human Nutrition, Wageningen University, Wageningen, The Netherlands; Universita degli Studi di Roma La Sapienza, ITALY

## Abstract

Cocoa consumption has beneficial cardiometabolic effects, but underlying mechanisms remain unclear. Epicatechin, the cocoa major monomeric flavan-3-ol, is considered to contribute to these cardio-protective effects. We investigated effects of pure epicatechin supplementation on gene expression profiles of immune cells in humans. In a double blind, placebo-controlled cross-over trial, 32 (pre)hypertensive subjects aged 30 to 80, received two 4-week interventions, i.e. epicatechin (100mg/day) or placebo with a 4-week wash-out between interventions. Gene expression profiles of peripheral blood mononuclear cells were determined before and after both interventions. Epicatechin regulated 1180 genes, of which 234 differed from placebo. Epicatechin upregulated gene sets involved in transcription and tubulin folding and downregulated gene sets involved in inflammation, PPAR signalling and adipogenesis. Several negatively enriched genes within these gene sets were involved in insulin signalling. Most inhibited upstream regulators within the epicatechin intervention were cytokines or involved in inflammation. No upstream regulators were identified compared to placebo. Epicatechin, a cocoa flavan-3-ol, reduces gene expression involved in inflammation, PPAR-signalling and adipogenesis in immune cells. Effects were mild but our findings increase our understanding and provide new leads on how epicatechin rich products like cocoa may affect immune cells and exert cardiometabolic protective effects.

## Introduction

Prospective cohort studies showed that higher levels of chocolate or cocoa consumption are associated with a lower risk of cardiometabolic disorders [1]. In addition, several randomized controlled intervention studies showed beneficial effects on intermediate markers of CVD, especially on blood pressure, endothelial function by flow mediated dilation (FMD), but also on insulin resistance [2–4]. Based on this evidence, the European Food and Safety Authority (EFSA) approved a health claim that cocoa flavan-3-ols help maintain normal endothelium-dependent vasodilation [5]. Although a cause-and-effect relationship has been established between cocoa/chocolate consumption and these cardiometabolic health markers, the underlying mechanisms are less well characterised in humans *in vivo*. Recently, we demonstrated in a human intervention study that 4-week daily consumption of dark chocolate improved FMD and also lowered adhesion molecules on leukocytes in healthy overweight middle aged men [6]. These findings may suggest a role of circulating immune cells in the cardio-protective effects of dark chocolate.

Cocoa is rich in flavan-3-ols. Epicatechin, its major monomeric flavan-3-ol, may be responsible for this beneficial effect on FMD [7]. However, chocolate contains a complex mixture of many flavan-3-ols and other substances, and the specific role of epicatechin is less well studied in isolation. For this reason, we recently conducted a human intervention study to determine the effects of pure epicatechin supplementation on markers of cardiometabolic health [8, 9]. Epicatechin supplementation did not change FMD significantly (1.1% absolute; 95% CI: -0.1%, 2.3%; P = 0.07), but findings from that study suggested that epicatechin may, in part, contribute to the cardio-protective effects of cocoa by lowering sE-selectin, a leukocyte adhesion molecule and marker of endothelial function, and by improving fasting insulin and insulin resistance. [8, 9]. This hypothesis is in line with an intervention study which showed that an improvement in endothelial function after 15 days of flavanol-rich dark chocolate consumption was paralleled by a decrease in insulin resistance [3].

Several *in vitro* experiments in human endothelial cells demonstrated that epicatechin stimulated the synthesis of nitric oxide (NO), an important mediator of vasodilation, and prevented the superoxide-mediated loss of NO [10, 11]. Other factors known to be important in endothelial health, such as inflammation, leukocyte adhesion and coagulation, might also be affected by epicatechin, but are less extensively studied in humans *in vivo* [6, 12, 13]. Peripheral blood mononuclear cells (PBMC) are immune cells that play an important role in inflammation and endothelial function and respond to changes in nutrient levels and inflammatory agents in blood. They are, therefore, valuable to study the cardio-protective mechanisms of epicatechin *in vivo* [14, 15]. A powerful strategy to understand how nutrients and bioactive compounds may affect cellular processes is by using whole genome wide screening techniques, such as transcriptomics. We demonstrated that atherosclerotic-related gene expression changes can be detected in PBMCs *in vivo* upon consumption of different types of fatty acids [14, 15]. Applying such high throughput screening tools may increase our understanding on how epicatechin may affect immune cells and thereby might exert its cardio-protective effects. For this reason, we aimed to investigate the effects of 4-week supplementation of pure epicatechin on whole genome gene expression profiles of PBMCs using a randomized double-blind, placebo-controlled crossover trial.

## Materials and methods

All subjects gave written informed consent. The study was approved by the Medical Ethics Committee of Wageningen and have been conducted according to the principles expressed in the Declaration of Helsinki. The study was registered at clinicaltrials.gov as NCT01691404.

### Subjects

We included samples from 37 Dutch individuals (25 men, 12 women) between 30–80 years old with untreated systolic BP levels between 125 and 160 mmHg from a previously reported study [8]. All subjects were non-smoking and did not have a disease.

### Study design

The original study [8, 9] investigated the effects of (-)-epicatechin and quercetin-3-glucoside in a randomized placebo controlled cross-over trial on markers of cardiometabolic health. Because epicatechin had more pronounced effects on these markers than quercetin, we selected only the epicatechin treatment for PBMCs transcriptome analysis and compared these effects with the placebo group.

The study was a randomized, double blind, placebo-controlled, cross-over trial in which received two 4-week interventions; epicatechin (100mg/day) or placebo (microcrystalline cellulose). Details of the study have been previously described [8]. In brief, subjects received both interventions in random order with a wash-out period of 4-weeks between both interventions. Subjects consumed 2 capsules per day with a glass of water: one during breakfast and one during dinner. Subjects were asked to avoid consumption of epicatechin-rich foods throughout the study. Fasting PBMCs were collected before (T0) and after each intervention period (T4). On the day prior to each study day, subjects received a standardized evening meal, refrained from alcohol consumption and strenuous exercise, and were not allowed to eat or drink anything except water after 10.00 pm.

### PBMC and RNA isolation

Fasting PBMCs were isolated before and after both intervention arms using BD Vacutainer Cell Preparation Tubes. RNA was isolated (RNeasy Micro kit, Qiagen, Venlo, the Netherlands), quantified (Nanodrop ND 1000, Nanodrop technologies, Wilmington, Delaware USA) and integrity was checked by an Agilent 2100 Bioanalyser with RNA 6000 microchips (Agilent Technologies, South Queensferry, UK). Samples were included for microarray analysis if the RNA integrity number (RIN) was > 7.

### Microarray processing

**Fig 1:**
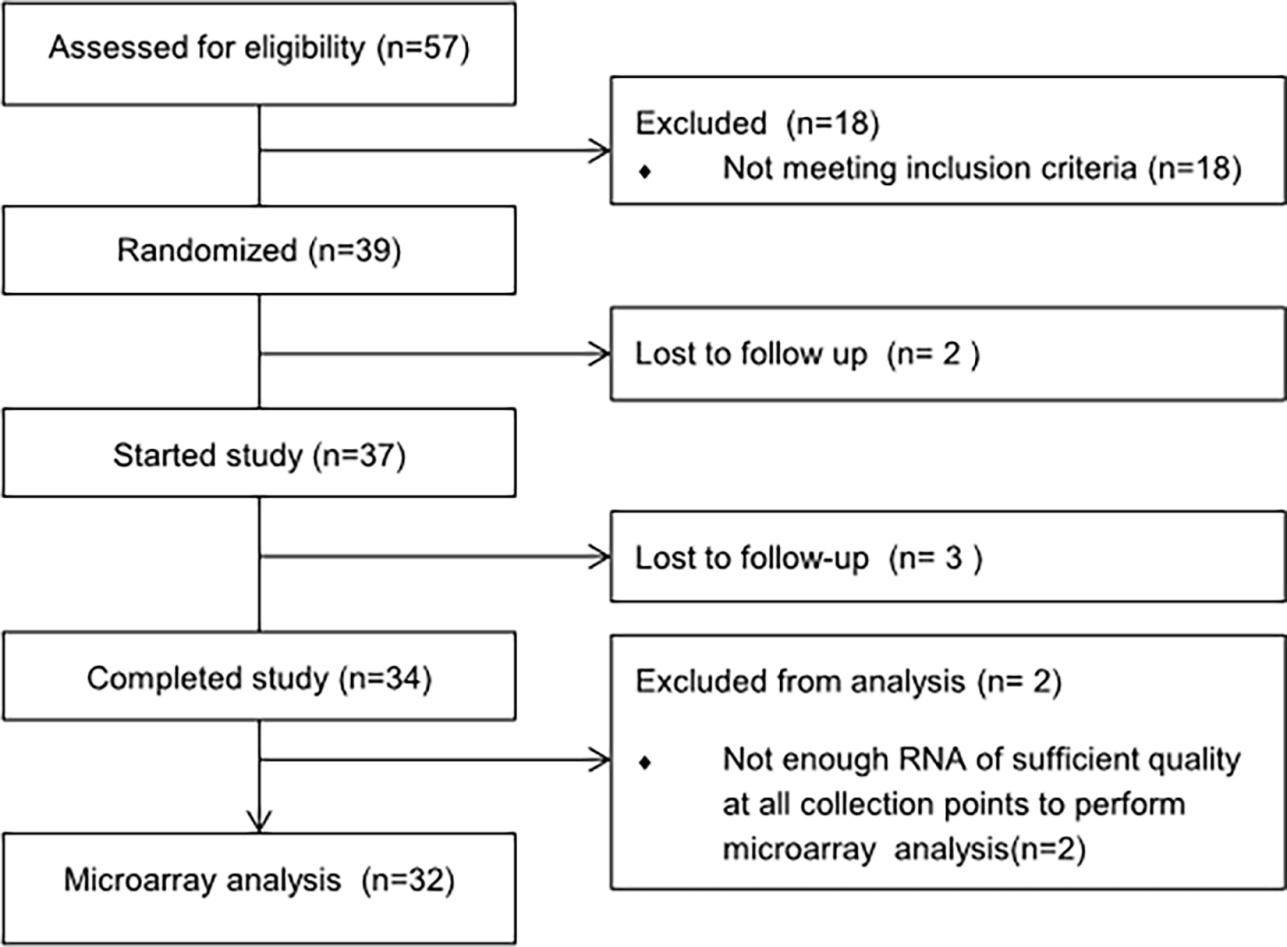
CONSORT flow diagram.

PBMC samples from 32 subjects yielded enough RNA of sufficient quality at all collection points to perform microarray analysis (Fig 1). Purified total RNA (100ng per sample) was labelled with the one-cycle cDNA labelling kit (MessageAmp™ II-Biotin Enhanced Kit; Ambion Inc, Nieuwekerk a/d IJssel, Netherlands) and hybridized to whole-genome Affymetrix GeneChip Human Gene 1.1 ST arrays (Affymetrix Inc. Santa Clara, CA). Sample labelling, hybridization to chips and image scanning were performed according to the manufacturers’ instructions.

### Microarray analysis

Microarray quality control and data analysis pipeline have been described in detail previously [16]. Briefly, normalized expression estimates of probe sets were computed by the robust multiarray analysis (RMA) algorithm [17, 18] as implemented in the Bioconductor library *AffyPLM*. Probe sets were redefined using current genome information according to Dai *et al*. [19] based on annotations provided by the Entrez Gene database, which resulted in the profiling of 19,654 unique genes (custom CDF v18). Genes having a normalized expression signal larger than 20 on more than 10 arrays were considered to be reliably expressed and selected for further analysis. Microarray data have been submitted to the Gene Expression Omnibus (accession number GSE84453).

Differentially expressed probe sets (genes) were identified by using linear models (package *limma*) and an intensity-based moderated t-statistic [20, 21]. By blocking on subject in the design matrix, the crossover design of the study was taken into account. The change over time in gene expression due to the intervention (Δ epicatechin or Δ placebo) or the difference between the change upon the epicatechin intervention and the placebo intervention (Δ epicatechin vs. Δ placebo) was considered significant if the *P*-value was <0.05. Changes in gene expression were related to changes in pathways by gene set enrichment analysis (GSEA) [22]. Gene sets were obtained from the KEGG, National Cancer Institute, Reactome and WikiPathways pathway databases. Only gene sets consisting of more than 10 and fewer than 500 genes were taken into account. The statistical significance of GSEA results was determined using 1,000 permutations. QIAGEN’s Ingenuity Pathway Analysis (IPA, QIAGEN Redwood City) was used to identify upstream regulators. Gene sets with a false discovery rate (FDR) Q-value <0.25 were defined as significantly regulated. An FDR of 0.25 indicates that the result is likely to be valid 3 out of 4 times, which is according to the GSEA user guide and reasonable in the setting of finding candidate hypothesis to be further validated as a results of future research. Upstream regulators were considered significant if *P*-value of overlap was <0.05.

## Results

**Fig 2:**
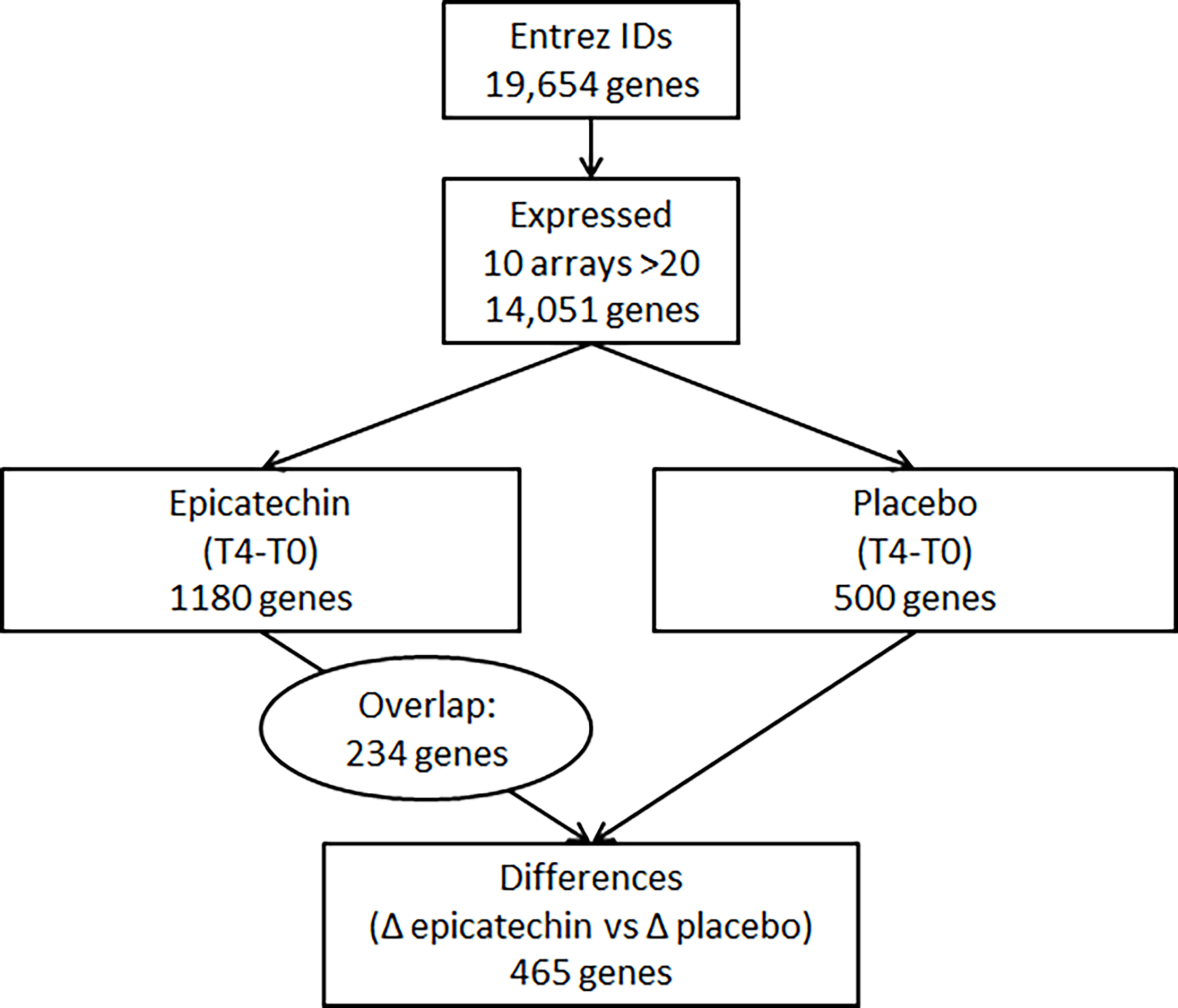
Flow diagram showing the number of genes of which the expression was changed after 4-week epicatechin or placebo supplementation and the number of genes that significantly changed in expression between the epicatechin or placebo intervention. A change was considered significant if two-sided *P*<0.05.

The mean age of the 32 subjects included in the microarray analysis was 65.8 ± 7.9 years and the mean BMI was 26.7 ± 3.5 kg/m^2^ (Table 1). Epicatechin supplementation significantly decreased plasma glucose and insulin and improved HOMA-IR and HOMA-β values (Table 2). Epicatechin supplementation changed the expression of 1180 genes (Δ epicatechin), and placebo changed the expression of 500 genes (Δplacebo) (Fig 2). Expression of 465 genes changed significantly different between the epicatechin and the placebo intervention (Δ epicatechin vs. Δ placebo). Of these 465 genes, 234 genes also changed in expression upon epicatechin intervention (Δ epicatechin) with an upregulated expression of 95 genes and a downregulated expression of 139 genes.

**Table 1:** Baseline characteristics of 32 untreated (pre)hypertensive healthy subjects included in the microarray analysis.

Characteristic	Value
Male/female	20/12
Age (yrs)	65.8 ± 7.82
BMI (kg/m^2^)	26.7 ± 3.52
Office SBP (mmHg)	128.2 ± 13.0
Office DBP (mmHg)	74.3 ± 9.3
Plasma glucose (mmol/L)	5.7 ± 0.6
Serum insulin (mU/L)	6.8 ± 4.5
HOMA-IR	1.7 ± 1.2
Fasting serum lipids (mmol/L)	
Total cholesterol	5.62 ± 0.75
LDL cholesterol	3.46 ± 0.65
HDL cholesterol	1.56 ± 0.44
Triglycerides	1.32 ± 0.51

Values are mean ± SD. Abbreviations: BMI, body mass index; DBP, diastolic blood pressure; HDL, high-density lipoprotein; HOMA-IR, homeostatic model assessment of insulin resistance; LDL, low-density lipoprotein; SBP, systolic blood pressure.

**Table 2:** Effects of epicatechin and placebo supplementation on markers of cardiometabolic health of the 32 participants included in the microarray analysis.

	Treatment effect		
	Epicatechin	Placebo	*P*-value
Body weight (kg)	0.17 ± 1.11	0.29 ± 1.78	0.75
Glucose (mmol/L)	-0.06 ± 0.38	-0.02 ± 0.35	0.03
Insulin (mU/L)	-0.80 ± 2.21	0.63 ± 2.37	0.02
HOMA-IR	-0.23 ± 0.66	0.15 ± 0.65	0.03
HOMA-β	-5.1 ± 17.6	6.3 ± 20.0	0.03
SBP (mmHg)	-3.04 ± 7.91	-2.59 ± 13.37	0.86
DBP (mmHg)	-0.90 ± 4.67	-0.88 ± 5.75	0.99

Values are mean ± SD. Abbreviations: DBP, diastolic blood pressure; HOMA-β, homeostatic model assessment of beta cell function; HOMA-IR, homeostatic model assessment of insulin resistance; SBP, systolic blood pressure.

### Gene set enrichment analysis

To elucidate which gene sets were regulated by epicatechin supplementation we performed a gene set enrichment analysis (GSEA). 193 gene sets were regulated within the epicatechin intervention and 33 in the placebo intervention. We identified in total 56 gene sets that were both significantly regulated between intervention arms (Δ epicatechin vs. Δ placebo) and within epicatechin intervention(Δ epicatechin) (Tables 3 and 4). 35 gene sets were upregulated and 21 were downregulated. Gene sets were clustered based on function or overlap in genes.

**Table 3:** Gene sets significantly upregulated within the epicatechin intervention (epicatechin) and between intervention arms (epicatechin versus placebo).

Gene set	SIZE	NES	FDRq	FDRq
	(# genes)		epicatechin	epicatechin versus placebo
**Transcription/translation**				
KEGG_RIBOSOME	74	2.0	0.14	0.23
REACT_FORMATION OF A POOL OF FREE 40S SUBUNITS	79	1.9	0.15	0.23
REACT_FORMATION OF THE TERNARY COMPLEX, AND SUBSEQUENTLY, THE 43S COMPLEX	41	1.9	0.12	0.25
REACT_POST-ELONGATION PROCESSING OF INTRONLESS PRE-MRNA	22	1.9	0.10	0.08
REACT_VIRAL MRNA TRANSLATION	68	1.9	0.09	0.24
REACT_EUKARYOTIC TRANSLATION TERMINATION	70	1.9	0.08	0.24
REACT_INFLUENZA VIRAL RNA TRANSCRIPTION AND REPLICATION	68	1.9	0.07	0.24
REACT_NONSENSE MEDIATED DECAY INDEPENDENT OF THE EXON JUNCTION COMPLEX	75	1.9	0.07	0.20
REACT_PROCESSING OF CAPPED INTRONLESS PRE-MRNA	22	1.9	0.06	0.09
WIP_HS_CYTOPLASMIC_RIBOSOMAL_PROTEINS	74	1.9	0.07	0.24
REACT_RNA POLYMERASE II TRANSCRIPTION TERMINATION	40	1.9	0.06	0.03
REACT_CLEAVAGE OF GROWING TRANSCRIPT IN THE TERMINATION REGION	40	1.8	0.06	0.08
REACT_POST-ELONGATION PROCESSING OF THE TRANSCRIPT	40	1.8	0.06	0.04
REACT_GENE EXPRESSION	492	1.8	0.06	0.12
REACT_GENERIC TRANSCRIPTION PATHWAY	204	1.8	0.08	0.25
REACT_POST-ELONGATION PROCESSING OF INTRON-CONTAINING PRE-MRNA	31	1.8	0.08	0.10
REACT_MRNA 3-END PROCESSING	31	1.8	0.08	0.13
REACT_3 -UTR-MEDIATED TRANSLATIONAL REGULATION	88	1.7	0.08	0.25
REACT_RNA POLYMERASE II TRANSCRIPTION	92	1.7	0.08	0.17
REACT_NONSENSE MEDIATED DECAY ENHANCED BY THE EXON JUNCTION COMPLEX	92	1.6	0.13	0.20
REACT_NONSENSE-MEDIATED DECAY	92	1.6	0.15	0.23
REACT_FORMATION AND MATURATION OF MRNA TRANSCRIPT	146	1.5	0.17	0.23
REACT_TRANSCRIPTION	131	1.5	0.18	0.23
REACT_METABOLISM OF NON-CODING RNA	21	1.5	0.20	0.24
**Tubulin folding**				
REACT_FORMATION OF TUBULIN FOLDING INTERMEDIATES BY CCT_TRIC	19	1.8	0.06	0.23
REACT_COOPERATION OF PREFOLDIN AND TRIC_CCT IN ACTIN AND TUBULIN FOLDING	26	1.8	0.06	0.24
REACT_PREFOLDIN MEDIATED TRANSFER OF SUBSTRATE TO CCT_TRIC	25	1.7	0.07	0.24
**Insulin**				
REACT_INSULIN SYNTHESIS AND PROCESSING	109	1.9	0.11	0.19
**Other**				
REACT_PD-1 SIGNALING	25	1.6	0.13	0.24
KEGG_ASTHMA	19	1.6	0.13	0.23
REACT_PHOSPHORYLATION OF CD3 AND TCR ZETA CHAINS	22	1.6	0.16	0.23
REACT_INTERACTIONS OF REV WITH HOST CELLULAR PROTEINS	32	1.6	0.16	0.24
REACT_NUCLEAR IMPORT OF REV PROTEIN	31	1.5	0.18	0.24
REACT_TRANSLOCATION OF ZAP-70 TO IMMUNOLOGICAL SYNAPSE	20	1.5	0.20	0.24
NCI_DNAPK_PATHWAY	15	1.5	0.20	0.25

Ranking based on normalised enrichment score (NES). FDRQ<0.25 was considered significant, 1116 gene sets were used in the analysis. Abbreviations: KEGG, Kyoto Encyclopedia of Genes and Genomes database; NCI, Nature Pathway Interaction database; REACT, Reactome knowledgebase; WIP_HS, WikiPathways Homo Sapiens.

**Table 4:** Gene sets significantly downregulated within the epicatechin intervention (epicatechin) and between intervention arms (epicatechin versus placebo).

Gene set	SIZE	NES	FDRq	FDRq
	(# genes)		epicatechin	epicatechin versus placebo
**Inflammation**				
NCI_IL8CXCR2_PATHWAY	31	-1.9	0.05	0.22
NCI_IL8CXCR1_PATHWAY	25	-1.9	0.04	0.18
NCI_AMB2_NEUTROPHILS_PATHWAY	30	-1.8	0.06	0.23
**PPAR**				
KEGG_PPAR SIGNALING PATHWAY	41	-1.6	0.15	0.21
REACT_REGULATION OF LIPID METABOLISM BY PPAR	41	-1.5	0.21	0.23
**GTPase**				
REACT_SIGNALING BY RHO GTPASES	102	-2.0	0.06	0.25
REACT_RHO GTPASE CYCLE	102	-1.9	0.04	0.21
NCI_RAC1_REG_PATHWAY	32	-1.9	0.06	0.22
NCI_CDC42_REG_PATHWAY	26	-1.5	0.24	0.23
**AMPK**				
WIP_HS_AMPK_SIGNALING	54	-1.6	0.17	0.22
**Other**				
WIP_HS_NOTCH_SIGNALING_PATHWAY	42	-2.3	0.00	0.22
NCI_RETINOIC_ACID_PATHWAY	23	-2.2	0.01	0.23
NCI_AURORA_A_PATHWAY	29	-2.0	0.05	0.24
WIP_HS_ADIPOGENESIS	103	-1.9	0.06	0.21
WIP_HS_FOLATE_METABOLISM	44	-1.8	0.09	0.23
NCI_HES_HEYPATHWAY	40	-1.7	0.13	0.24
NCI_AP1_PATHWAY	55	-1.6	0.16	0.23
WIP_HS_ANGIOGENESIS	17	-1.6	0.16	0.24
REACT_TRANSMISSION ACROSS CHEMICAL SYNAPSES	103	-1.5	0.20	0.23
REACT_SYNAPTIC TRANSMISSION	143	-1.5	0.20	0.22
REACT_G ALPHA (Q) SIGNALLING EVENTS	106	-1.4	0.25	0.22

Ranking based on normalised enrichment score (NES). FDRQ<0.25 was considered significant, 1116 gene sets were used in the analysis. Abbreviations: KEGG, Kyoto Encyclopedia of Genes and Genomes database; NCI, Nature Pathway Interaction database; REACT, Reactome knowledgebase; WIP_HS, WikiPathways Homo Sapiens.

#### Upregulated gene sets

Of the upregulated gene sets, one cluster was involved in in transcription and contributing genes within this gene set cluster largely included zinc finger proteins and ribosomal proteins (S1 Fig). Another cluster included genes involved in ‘tubulin folding’. In the current study we observed that epicatechin supplementation resulted in improved fasting insulin and insulin resistance [8]. We therefore specifically investigated if related pathways were affected. The insulin synthesis and processing pathway was upregulated after the epicatechin intervention and between intervention arms (Δ epicatechin vs. Δ placebo) (Table 3). However, contributing genes within the insulin synthesis and processing pathway were mainly ribosomal proteins and largely overlapped with the transcription/translation cluster (S1 Fig). No genes specifically involved in insulin synthesis were upregulated.

#### Downregulated gene sets

**Fig 3:**
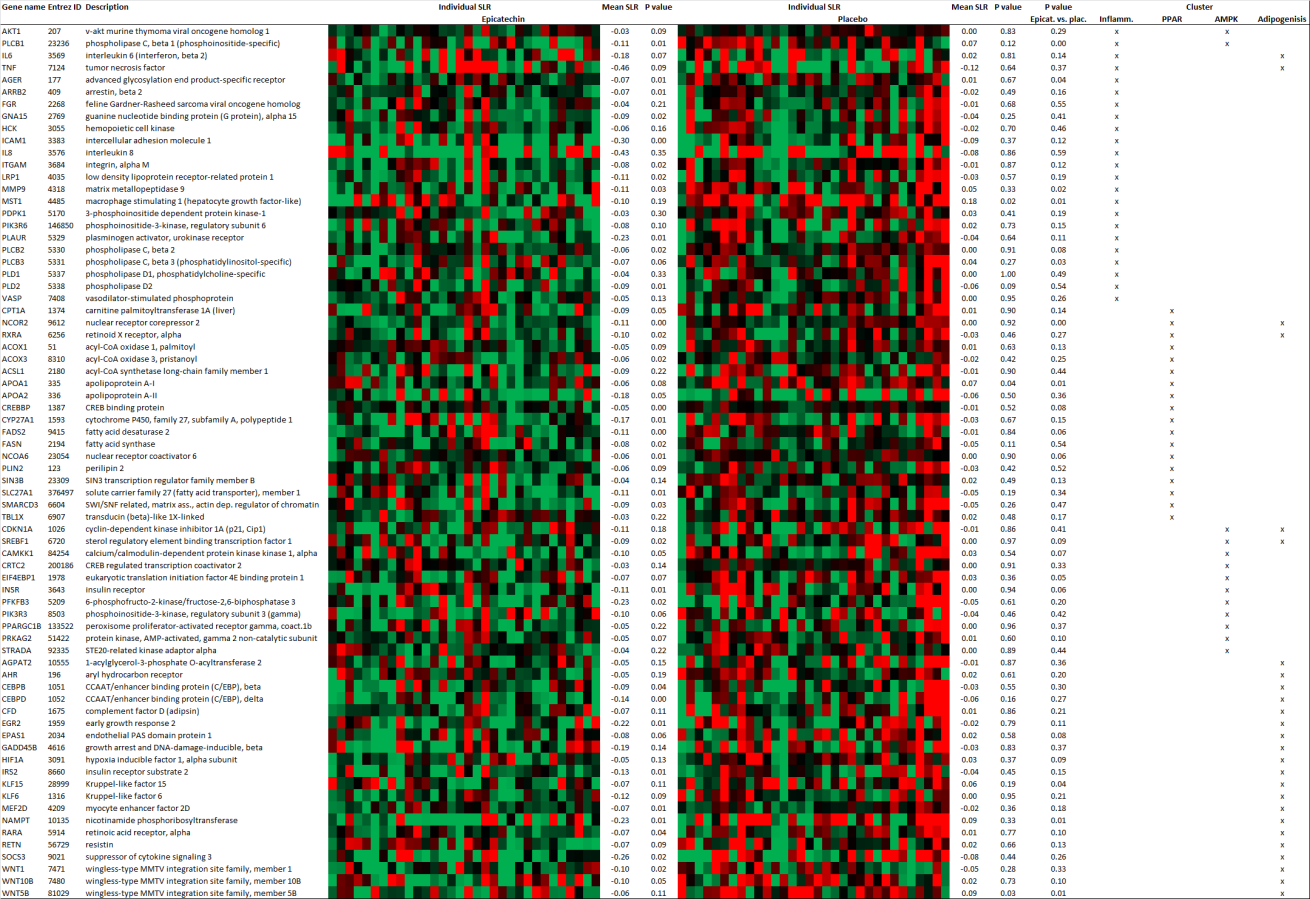
Expression heat map of contributing genes within the downregulated gene sets involved in inflammation, PPAR signalling, AMPK signalling and adipogenesis. Subjects were hierarchically clustered via the complete agglomeration method. Expression changes are indicated as individual signal-log-ratios (SLR) of T = 4 weeks versus T = 0 weeks. Down-regulation or up-regulation of gene expression is presented on a colour scale, ranging from green (downregulated, SLR ≤-0.25) to red (upregulated, SLR ≥ 0.25).

The downregulated clusters included inflammatory related gene sets, such as IL8–CXCR1/2 pathways and AMB2_neutrophil pathway, which are known to be related to vascular health. PPAR signalling, adipogenesis and the AMPK signalling pathway were also downregulated. Individual changes in expression of the contributing genes within the above described downregulated gene sets are depicted in a heat map (Fig 3). Contributing genes within the PPAR signalling gene set included genes involved in β-oxidation, such as *ACSL1*, *CPT1a* and *ACOX*. Interestingly the insulin receptor gene (*INSR*) was one of the downregulated contributing genes within the AMPK gene set. Also downstream targets of the insulin receptor, such as IRS2, AKT1 and PIK3R3 and the transcription factor *SREBF1* and its downstream targets *FASN* and *FADS2* were negatively enriched within the above described downregulated gene sets. These changes point towards an enriched downregulation of the insulin signalling pathway upon epicatechin supplementation.

### Upstream regulator analysis

To identify common upstream regulators of the affected genes we performed an upstream regulator analysis using Ingenuity Pathway Analysis. No upstream regulators were identified if epicatechin was compared to placebo. When analysing the effects within the epicatechin supplementation group alone, 13 activated and 84 inhibited potential upstream regulators were identified (Table A in S1 File) and 2 upstream regulators were identified upon placebo supplementation (Table B in S1 File). No overlapping potential upstream regulators were identified between the two interventions. The majority of highly ranked inhibited upstream regulators upon epicatechin supplementation were classified as inflammatory type molecules (NF-kB, RELA (NF-kB p65 subunit), TLR4 and TLR7), platelet-derived growth factor complex (PDGF BB), or as a cytokine (TNF, CCL5, IL1B, IL6, IL5, IL1A, IL17A and IFNG).

### Correlations with measures of endothelial function and insulin

To identify potential associations between the changes in gene expression and changes in vascular health, the gene expression changes of the 234 genes significantly changed by epicatechin supplementation compared to placebo were correlated with changes in flow mediated dilation (FMD) and with changes in plasma insulin (Tables C and D in S1 File). Changes in FMD correlated significantly with the expression changes of 10 genes within the epicatechin intervention. Within those 234 genes, 15 genes correlated significantly in the placebo intervention. Intervention induced changes in plasma insulin correlated with changes in expression of 17 and 6 genes in the epicatechin and placebo group, respectively. No significant correlations were observed if we applied a false discovery rate correction for multiple testing.

## Discussion

In a 4-week randomised, double-blind, placebo-controlled crossover trial we demonstrated that epicatechin supplementation affected PBMC gene expression profiles. Epicatechin downregulated gene sets involved in inflammation, PPAR signalling and adipogenesis and upregulated gene sets involved in transcription/translation and tubulin folding.

The down-regulation of the inflammation-related cluster IL8 –CXCR1/2 and AMB2_neutrophil pathway points in the direction of an anti-inflammatory effect by epicatechin. Furthermore, a number of upstream regulators, including NF-κB, TNF and IL1b were predicted to be inhibited upon epicatechin supplementation. These latter finding did not remain, however, when compared to placebo. If epicatechin does exert anti-inflammatory effects, we hypothesise that these effects may be mediated via inhibition of inflammatory transcription factors such as NF-κB and AP-1 [23]. This hypothesis is supported by a previously published human intervention trial which showed that cocoa powder (28mg epicatechin) significantly decreased NF-κB activation in PBMCs compared to baseline [24]. Similarly, Morrison et al. showed that pure epicatechin supplementation prevented diet-induced activation of aortic NF-κB in ApoE*3-Leiden mice fed an atherogenic diet [25]. A potential reduction in expression of genes involved in inflammation by epicatechin may be one of the contributing factors to the cardio-protective effects of cocoa [26]. Yet, the effects of epicatechin supplementation on plasma markers of endothelial dysfunction or inflammation in this intervention study were relatively mild; out of 11 plasma markers of inflammation or endothelial dysfunction, only sE-selectin was decreased by epicatechin supplementation [9]. However, changes in plasma markers reflect a systemic response to epicatechin coming from several tissues. Because the current transcriptome analysis was performed in circulating immune cells, plasma markers do not need to reflect changes in the circulating immune cells.

PPAR signalling and adipogenesis, both regulators of in lipid metabolism, were inhibited upon epicatechin supplementation. *In vitro* studies also report that cocoa polyphenol extracts can suppress adipogenesis in preadipocytes [27] and that similar type molecules, such as (-)-epigallocatechin-3-gallate, can have inhibitory effects on lipid accumulation and adipogenesis in these cells [28].Findings on PPAR signalling are not consistent as other *in vitro* and animal studies reported that cocoa related polyphenols may activate and not inhibit PPAR signaling [23]. We could not find other human studies investigating the effect of epicatechin on whole genome gene expression, but we previously conducted a randomized intervention trial with other types of polyphenols, namely isoflavones [29]. In that study the expression of PPARα in PBMCs was also significantly decreased after 8-wk exposure to isoflavones compared to placebo. In the current study we supplemented our subjects with pure epicatechin, whereas others tested mixtures or other types of polyphenols. Small differences in chemical structure are known to have a big impact on bio-availability and efficacy [30, 31] and hence may explain the opposite effects observed in our study versus the *in vitro* and animal studies. Another explanation for this opposite effect of pure epicatechin on PPAR activation between our study and previous *in vitro* and animal studies may be that the direction of transcriptional activity is dependent on dose. Such biphasic properties have already been described *in vitro* for the isoflavones genistein and daidzein by Dang *et al*. [32, 33]. Effects of polyphenols on PPAR activation can also be different between tissues and species [34].

We previously reported that epicatechin supplementation decreased plasma insulin and improved insulin resistance in this study [8]. These results were upheld in our subgroup included in the microarray analysis. Interestingly, several contributing genes within the downregulated gene sets AMPK and adipogenesis were involved in insulin signalling, including the insulin receptor and several downstream targets. Perhaps this downregulation in expression of insulin signalling related genes was driven by the decrease in plasma insulin levels observed in this study. However, it needs to be noted that these genes were enriched but not significantly regulated between intervention arms and within epicatechin intervention despite the relatively large number of subjects. As insulin signalling is especially of relevance in metabolically active organs, such as muscle, immune cells may not be the optimal cell-type to study the observed effects of epicatechin on insulin resistance. In addition, many changes in insulin signalling are regulated on the protein level by, for instance by phosphorylation [35].

Cardiometabolic health effects of pure epicatechin may also work via an increased NO bioavailability [36]. A hypothesized mechanism through which epicatechin may increase the bioavailability of NO is by protecting against oxidative damage via the transcription factor Nrf2 [37]. However, our gene expression and upstream regulator analyses results do not provide support in this direction. To further explore potential association between changes in PBMCs gene expression and other outcome measures known to be affected by epicatechin, a correlation analyses was performed. However, the few correlations found upon epicatechin supplementation were weak and can be classified as false-positives considering that these effects were not significant after a false discovery rate correction and by the fact that the amount of correlations observed after placebo supplementation was similar to the number of significant correlations observed after epicatechin supplementation.

Besides these potential direct effects of epicatechin, it may also be that epicatechin derived metabolites may contribute to the observed effects. A large proportion epicatechin is not absorbed in the small intestine, but passes through to the large intestine where resident bacteria generate microbial metabolites. As these epicatechin derived metabolites can appear in the circulation [38, 39] they may have potential beneficial health effects [40, 41].

The compliance in this relatively large cross-over study was high. Over 98% of the distributed supplements were consumed, causing a marked increase in plasma epicatechin concentrations upon acute consumption of the capsules [8]. Subjects were also weight stable during the intervention. We compared the changes induced by 4-week supplementation of epicatechin to those induced by placebo supplementation resulting in 234 changed genes without false discovery rate controlling procedures. We considered genes changed if they were significantly changed after both the epicatechin intervention and between the epicatechin and the placebo intervention. Such an approach is rather strict, but in our opinion it is also the most valid way of performing such analysis to enable elucidating the real epicatechin-induced effects. Despite the large number of subjects and the cross-over design, the number of genes of which the expression was found to be significantly changed by epicatechin supplementation compared to the placebo was smaller than observed for interventions with other nutrients such as fatty acids [15]. The large variation in individual responses in the epicatechin group and the variation of expression changes in the placebo group may explain this relatively low number. As a consequence, pure epicatechin, in concentrations achievable with dietary exposure, did not show robust effects on gene expression in immune cells compared to the placebo.

In humans, epicatechin is absorbed by epithelial cells in the jejunum and completely metabolised upon absorption to (-)-epicatechin glucuronides, sulfates, and O-methyl sulfates [42]. The absorption and conjugation patterns may differ substantially between subjects [7] and may, therefore, differently affect receptors and transcription factors, hence resulting in a large variation in gene expression response. Previous studies in our group on the effects of isoflavones demonstrated that the effects of isoflavones on whole-genome wide expression are more pronounced in adipose tissue [43] compared to PBMCs [29]. Similarly, the effect of epicatechin might be more pronounced in other cells, such as endothelial cells, adipose tissue or muscle tissue [44].

In conclusion, pure epicatechin a major flavan-3-ol from cocoa, inhibited gene expression of inflammation signalling routes, PPAR signalling, adipogenesis and insulin signalling in circulating immune cells from (pre)hypertensive men and women. Effects were relatively mild but these findings increase our understanding and provide new leads on how epicatechin-rich products such as cacao may affect immune cells and thereby might exert its cardiometabolic protective effects.

## Supporting information

S1 Checklist (PDF)

S1 Fig Expression heat map of contributing genes within the upregulated gene sets involved in transcription/translation, tubulin folding and insulin synthesis and processing. Subjects were hierarchically clustered via the complete agglomeration method. Expression changes are indicated as individual signal-log-ratios (SLR) of T = 4 weeks versus T = 0 weeks. Downregulation or upregulation of gene expression is presented on a colour scale, ranging from green (downregulated, SLR ≤-0.25) to red (upregulated, SLR ≥ 0.25).
(TIF)

S1 File Tables A-D.
(PDF)

S1 Protocol (PDF)

## Abbreviations

EFSA: European Food and Safety Authority
FDR: false discovery rate
FMD: flow mediated dilation
GSEA: gene set enrichment analysis
NO: nitric oxide
PBMC: peripheral blood mononuclear cells
RMA: robust multichip average
SLR: signal-to-log ratios
