# Supplementary figures and images for: Pure flavonoid epicatechin and whole genome gene expression profiles in circulating immune cells in adults with elevated blood pressure: A randomised double-blind, placebo-controlled, crossover trial
Source: PLoS One. 2018 Apr 19;13(4):e0194229. doi: 10.1371/journal.pone.0194229 (PMC5908087; doi:10.1371/journal.pone.0194229)

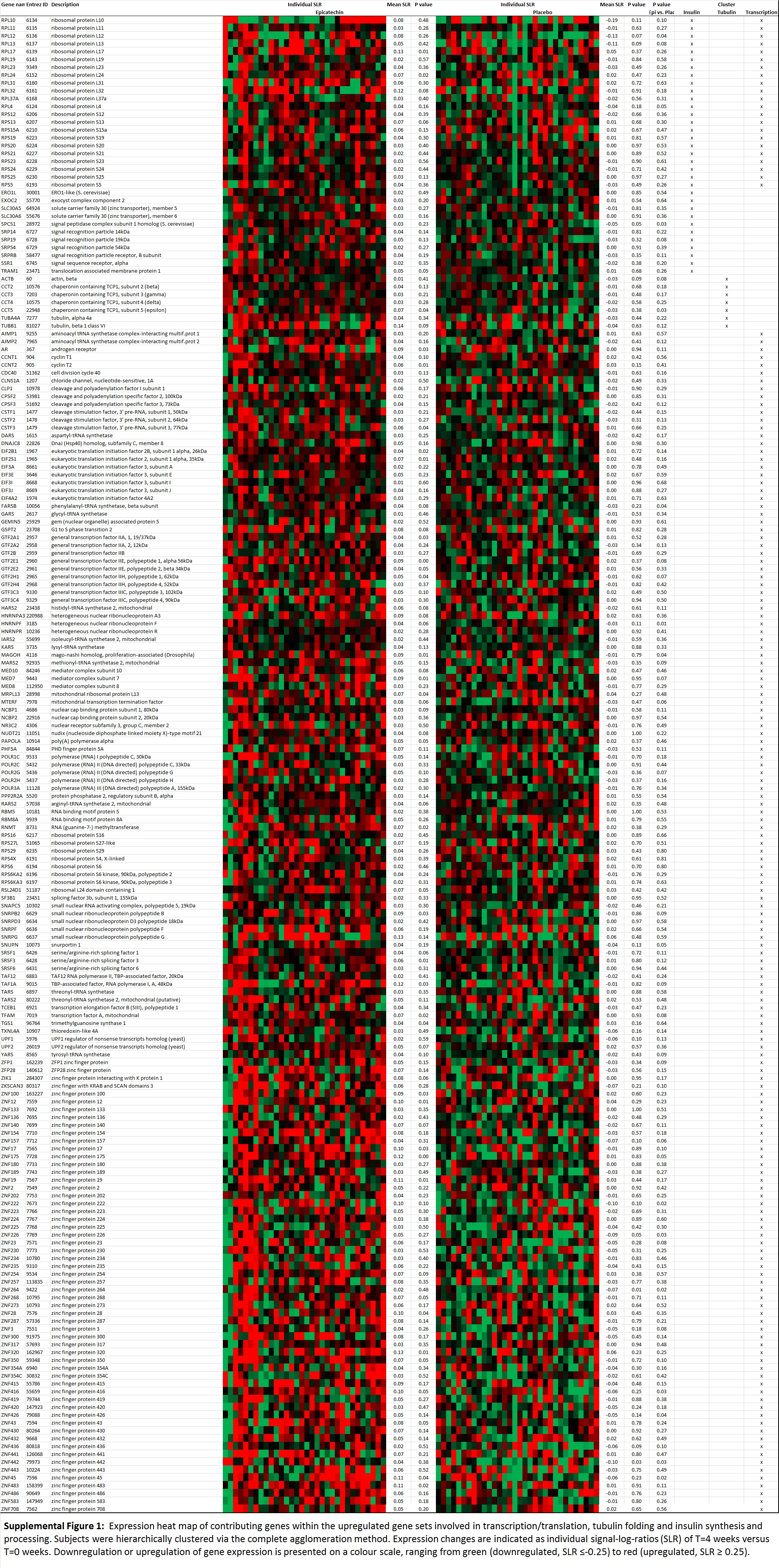

Supplement: S1 Fig — Subjects were hierarchically clustered via the complete agglomeration method. Expression changes are indicated as individual signal-log-ratios (SLR) of T = 4 weeks versus T = 0 weeks. Downregulation or upregulation of gene expression is presented on a colour scale, ranging from green (downregulated, SLR ≤-0.25) to red (upregulated, SLR ≥ 0.25). (TIF) [file pone.0194229.s002.tif]
