# Supplementary material for: Pure flavonoid epicatechin and whole genome gene expression profiles in circulating immune cells in adults with elevated blood pressure: A randomised double-blind, placebo-controlled, crossover trial
Source: PLoS One. 2018 Apr 19;13(4):e0194229. doi: 10.1371/journal.pone.0194229 (PMC5908087; doi:10.1371/journal.pone.0194229)
Supplement: S1 File — Tables A-D. (PDF) [file pone.0194229.s003.pdf]

**Table A.** Predicted upstream regulators upon epicatechin supplementation (Δ epicatechin).

| Upstream Regulator | Molecule Type | Predicted Activation State | Activation z-score | P-value of Overlap |
|---|---|---|---|---|
| NFkB (complex) | complex | Inhibited | -5.38 | <0.01 |
| PDGF BB | complex | Inhibited | -5.18 | <0.01 |
| TNF | cytokine | Inhibited | -5.03 | <0.01 |
| EGF | growth factor | Inhibited | -4.59 | 0.01 |
| IL1B | cytokine | Inhibited | -4.35 | <0.01 |
| NUPR1 | transcription regulator | Inhibited | -4.35 | <0.01 |
| EGFR | Kinase | Inhibited | -4.25 | <0.01 |
| TGFB1 | growth factor | Inhibited | -4.17 | <0.01 |
| F2 | peptidase | Inhibited | -4.14 | 0.01 |
| Cg | complex | Inhibited | -3.98 | <0.01 |
| TLR4 | transmembrane receptor | Inhibited | -3.96 | <0.01 |
| STAT3 | transcription regulator | Inhibited | -3.81 | <0.01 |
| IL6 | cytokine | Inhibited | -3.67 | 0.01 |
| IL17A | cytokine | Inhibited | -3.52 | <0.01 |
| IGF1 | growth factor | Inhibited | -3.47 | 0.04 |
| ERBB2 | Kinase | Inhibited | -3.47 | <0.01 |
| CSF3 | cytokine | Inhibited | -3.43 | <0.01 |
| RELA | transcription regulator | Inhibited | -3.34 | <0.01 |
| IFNG | Cytokine | Inhibited | -3.33 | <0.01 |
| MAP2K1 | Kinase | Inhibited | -3.27 | 0.02 |
| CSF2 | cytokine | Inhibited | -3.19 | <0.01 |
| MYD88 | Other | Inhibited | -3.19 | <0.01 |
| SP1 | transcription regulator | Inhibited | -3.19 | <0.01 |
| IL5 | cytokine | Inhibited | -3.18 | <0.01 |
| IL1A | cytokine | Inhibited | -3.18 | 0.04 |
| CREM | transcription regulator | Inhibited | -3.15 | 0.03 |
| HMGB1 | transcription regulator | Inhibited | -3.08 | <0.01 |
| ADIPOQ | Other | Inhibited | -3.05 | 0.02 |
| SYVN1 | transporter | Inhibited | -3.05 | 0.02 |
| NRG1 | Other | Inhibited | -2.98 | <0.01 |
| TLR7 | transmembrane receptor | Inhibited | -2.95 | 0.04 |
| PGR | ligand-dependent nuclear receptor | Inhibited | -2.84 | 0.04 |
| CSF1 | cytokine | Inhibited | -2.82 | <0.01 |
| EDN1 | cytokine | Inhibited | -2.81 | 0.01 |
| FOXO1 | transcription regulator | Inhibited | -2.81 | <0.01 |
| FOXL2 | transcription regulator | Inhibited | -2.81 | <0.01 |
| Creb | Group | Inhibited | -2.78 | 0.01 |
| EIF2AK2 | Kinase | Inhibited | -2.76 | 0.01 |
| CAMP | Other | Inhibited | -2.75 | <0.01 |
| TICAM1 | Other | Inhibited | -2.75 | <0.01 |
| IKBKB | Kinase | Inhibited | -2.66 | <0.01 |

| | | | | |
|---|---|---|---|---|
| EGR1 | transcription regulator | Inhibited | -2.65 | 0.01 |
| TGM2 | enzyme | Inhibited | -2.64 | <0.01 |
| TREM1 | transmembrane receptor | Inhibited | -2.61 | <0.01 |
| IKBKG | Kinase | Inhibited | -2.61 | <0.01 |
| NOD2 | Other | Inhibited | -2.60 | 0.02 |
| TNFSF13B | cytokine | Inhibited | -2.59 | 0.01 |
| Fibrinogen | complex | Inhibited | -2.57 | <0.01 |
| PI3K (complex) | complex | Inhibited | -2.50 | <0.01 |
| LDL | complex | Inhibited | -2.48 | <0.01 |
| TNFSF11 | cytokine | Inhibited | -2.46 | <0.01 |
| ECSIT | transcription regulator | Inhibited | -2.45 | <0.01 |
| AGT | growth factor | Inhibited | -2.45 | 0.04 |
| CHUK | kinase | Inhibited | -2.44 | <0.01 |
| IFNGR1 | transmembrane receptor | Inhibited | -2.43 | 0.01 |
| TRADD | other | Inhibited | -2.43 | <0.01 |
| IL32 | cytokine | Inhibited | -2.42 | 0.03 |
| ID3 | transcription regulator | Inhibited | -2.40 | <0.01 |
| TLR5 | transmembrane receptor | Inhibited | -2.39 | 0.01 |
| PLAUR | transmembrane receptor | Inhibited | -2.38 | 0.01 |
| CCL2 | cytokine | Inhibited | -2.37 | <0.01 |
| C5 | cytokine | Inhibited | -2.35 | <0.01 |
| JUN | transcription regulator | Inhibited | -2.34 | 0.01 |
| TGFA | growth factor | Inhibited | -2.34 | 0.01 |
| CD5 | transmembrane receptor | Inhibited | -2.33 | <0.01 |
| P38 MAPK | group | Inhibited | -2.32 | 0.02 |
| IRF8 | transcription regulator | Inhibited | -2.29 | <0.01 |
| FOS | transcription regulator | Inhibited | -2.24 | <0.01 |
| BTC | growth factor | Inhibited | -2.24 | <0.01 |
| CFB | peptidase | Inhibited | -2.22 | <0.01 |
| IL13 | cytokine | Inhibited | -2.22 | 0.01 |
| NCR2 | transmembrane receptor | Inhibited | -2.21 | <0.01 |
| CCL5 | cytokine | Inhibited | -2.21 | <0.01 |
| Growth hormone | group | Inhibited | -2.21 | <0.01 |
| Pro-inflam.Cytokine | group | Inhibited | -2.20 | 0.03 |
| JUND | transcription regulator | Inhibited | -2.19 | <0.01 |
| ITGA5 | transmembrane receptor | Inhibited | -2.18 | 0.02 |
| S100A4 | other | Inhibited | -2.17 | 0.01 |
| EIF4E | translation regulator | Inhibited | -2.14 | 0.01 |
| RETN | other | Inhibited | -2.10 | <0.01 |
| Vegf | group | Inhibited | -2.09 | 0.01 |
| ERK1/2 | group | Inhibited | -2.09 | <0.01 |
| MAPK14 | kinase | Inhibited | -2.05 | <0.01 |
| CEBPD | transcription regulator | Inhibited | -2.04 | 0.04 |
| LGALS3 | other | Activated | 2.01 | 0.03 |
| FLT1 | kinase | Activated | 2.12 | 0.03 |
| miR-155-5p | mature microrna | Activated | 2.17 | <0.01 |

| | | | | |
|---|---|---|---|---|
| miR-27a-3p | mature microrna | Activated | 2.22 | 0.04 |
| SOX7 | transcription regulator | Activated | 2.24 | 0.02 |
| IL1RN | cytokine | Activated | 2.38 | 0.02 |
| miR-34a-5p | mature microrna | Activated | 2.43 | 0.04 |
| miR-124-3p | mature microrna | Activated | 2.54 | 0.02 |
| Alpha catenin | group | Activated | 2.55 | <0.01 |
| GFI1 | transcription regulator | Activated | 2.65 | 0.02 |
| GPX1 | enzyme | Activated | 2.80 | <0.01 |
| DUSP1 | phosphatase | Activated | 2.88 | <0.01 |
| ESR1 | ligand-dependent nuclear receptor | Activated | 3.92 | <0.01 |

Upstream regulators were considered predicted if two-sided *P* value <0.05 and activation z-score > 2 or < -2. Upstream regulators classified as chemicals/drugs were excluded.

**Table B.** Predicted upstream regulators upon placebo supplementation (Δ placebo).

| Upstream Regulator | Molecule Type | Predicted Activation State | Activation z-score | p-value of overlap |
|---|---|---|---|---|
| ERBB3 | kinase | Inhibited | -2.22 | 0.03 |
| CD24 | other | Activated | 2.00 | 0.02 |

Upstream regulators were considered predicted if two-sided *P* value <0.05 and activation z-score > 2 or < -2. Upstream regulators classified as chemicals/drugs were excluded.

**Table C.** Genes of which expression changes showed significant correlation with changes in FMD after the epicatechin or placebo intervention

| | Epicatechin | | Placebo | |
|---|---|---|---|---|
| Gene | *r* | *P*-value | *r* | *P*-value |
| *RRAGA* | 0.52 | 0.01 | -0.08 | NS |
| *VPS29* | 0.48 | 0.02 | 0.24 | NS |
| *RPL9* | -0.46 | 0.02 | -0.07 | NS |
| *FBXO39* | -0.45 | 0.03 | -0.20 | NS |
| *LOC100128288* | -0.45 | 0.03 | -0.30 | NS |
| *ARID1B* | -0.44 | 0.03 | -0.10 | NS |
| *SLC25A35* | -0.43 | 0.04 | 0.25 | NS |
| *SUMO1* | 0.43 | 0.04 | 0.32 | NS |
| *ST8SIA1* | 0.42 | 0.04 | -0.31 | NS |
| *USP51* | 0.42 | 0.04 | 0.16 | NS |
| *HRK* | 0.23 | NS | 0.56 | 0.00 |
| *ARHGAP10* | -0.07 | NS | -0.47 | 0.01 |
| *FKBP9* | -0.08 | NS | 0.45 | 0.02 |
| *FAM149B1* | -0.22 | NS | 0.45 | 0.02 |
| *EEPD1* | 0.13 | NS | 0.44 | 0.02 |
| *GFI1* | -0.23 | NS | -0.42 | 0.03 |
| *CCDC149* | -0.09 | NS | 0.42 | 0.03 |
| *MAGOH* | -0.17 | NS | 0.41 | 0.03 |
| *NFIA* | -0.04 | NS | 0.41 | 0.03 |
| *SLITRK4* | -0.01 | NS | 0.39 | 0.04 |
| *C2orf48* | -0.04 | NS | 0.39 | 0.04 |
| *CD180* | 0.08 | NS | 0.38 | 0.04 |
| *NAMPT* | 0.00 | NS | 0.38 | 0.04 |
| *BIK* | 0.24 | NS | 0.38 | 0.05 |
| *OR10G4* | -0.06 | NS | -0.38 | 0.05 |

Pearson Correlation was performed on signal-to-log ratios (SLR). Correlations were performed on 234 genes significantly regulated by epicatechin supplementation compared to the placebo. Depicted correlations were significant in the epicatechin (*n*=24) or placebo (*n*=28) intervention. NS: not significant.

**Table D.** Genes of which expression changes showed significant correlation with changes in insulin after the epicatechin or placebo intervention

| | Epicatechin | | Placebo | |
|---|---|---|---|---|
| Gene | *r* | *P*-value | *r* | *P*-value |
| *JMJD6* | 0.44 | 0.01 | 0.11 | NS |
| *PLXNA1* | -0.42 | 0.02 | 0.00 | NS |
| *MT1M* | 0.39 | 0.03 | -0.19 | NS |
| *LINC00910* | -0.39 | 0.03 | -0.09 | NS |
| *MUT* | 0.39 | 0.03 | 0.15 | NS |
| *LRCH4* | -0.38 | 0.03 | -0.28 | NS |
| *ARMC5* | -0.38 | 0.03 | -0.07 | NS |
| *EGLN3* | 0.38 | 0.03 | -0.14 | NS |
| *CNFN* | -0.38 | 0.03 | -0.27 | NS |
| *DCK* | 0.38 | 0.03 | 0.25 | NS |
| *MYPOP* | -0.37 | 0.04 | 0.22 | NS |
| *CES4A* | 0.37 | 0.04 | 0.07 | NS |
| *FOXQ1* | -0.37 | 0.04 | -0.09 | NS |
| *OR10G4* | -0.37 | 0.04 | -0.25 | NS |
| *AGXT* | -0.36 | 0.04 | 0.02 | NS |
| *PLXND1* | -0.36 | 0.04 | -0.05 | NS |
| *KRBA1* | 0.36 | 0.04 | -0.16 | NS |
| *MIER3* | 0.14 | NS | 0.46 | 0.01 |
| *MAN2C1* | -0.27 | NS | -0.46 | 0.01 |
| *RAE1* | 0.13 | NS | 0.42 | 0.02 |
| *PQLC2* | 0.03 | NS | -0.41 | 0.02 |
| *SHISA7* | -0.11 | NS | -0.41 | 0.02 |
| *BNIP1* | -0.10 | NS | 0.37 | 0.04 |

Pearson Correlation was performed on signal-to-log ratios (SLR). Correlations were performed on 234 genes significantly regulated by epicatechin supplementation compared to the placebo. Depicted correlations were significant in the epicatechin or placebo (*n*=32) intervention. NS: not significant.
