# Supplementary material for: Pure flavonoid epicatechin and whole genome gene expression profiles in circulating immune cells in adults with elevated blood pressure: A randomised double-blind, placebo-controlled, crossover trial
Source: PLoS One. 2018 Apr 19;13(4):e0194229. doi: 10.1371/journal.pone.0194229 (PMC5908087; doi:10.1371/journal.pone.0194229)
Supplement: S1 Protocol — (PDF) [file pone.0194229.s004.pdf]

RESEARCH PROTOCOL

Version 2 – June 2012

Relevance of Vascular Function Markers: Short-term Intervention on the Effects of Quercetin, Epicatechin and High-Flavanol Cocoa on Vascular Function in (Pre)Hypertensive Subjects

The FLAVO-studie

James Dower, MSc

Dr. ir. Peter Hollman

A collaboration of

Top Institute Food and Nutrition and

Division of Human Nutrition, Wageningen

TI FOOD NUTRITION

WAGENINGEN UNIVERSITY
WAGENINGEN UR

PROTOCOL TITLE : Relevance of Vascular Function Markers - Short-term Intervention on the Effects of Quercetin, Epicatechin and High-Flavanol Cocoa on Vascular Function in (Pre)Hypertensive Subjects

**Protocol ID**	FLAVO-studie
**Short title**	Effects of Pure Flavonoids on Cardiovascular Health
**Version**	2.0
**Date**	12-06-2112
**Coordinating investigator/project leader**	Dr. P.C.H. Hollman<br>Wageningen University, Division of Human Nutrition<br>Postbus 230<br>6700AE, Wageningen
**Principal investigator(s)**	James Dower<br>Wageningen University, Division of Human Nutrition<br>Postbus 8129,<br>6700 EV, Wageningen<br>Tel: +31 (0)317 481486<br>email: james.dower@wur.nl
**Sponsor (in Dutch: verrichter/opdrachtgever)**	Top Institute Food and Nutrition (TIFN)<br>P.O. Box 557<br>6700 AN, Wageningen
**Independent physician**	Dr. J.J. van Binsbergen<br>Voorstraat 71, 3231 BK, Brielle<br>Tel: 0181-412155<br>email: j.vanbinsbergen@elg.umcn.nl
**Study physician**	Dr. M.R. Mensink<br>P.O. Box 8129, 6700 EV Wageningen<br>Tel: +31 (0)317-482646<br>email: marco.mensink@wur.nl
**Laboratory sites**	Department of Internal Medicine<br>Maastricht University<br>Universiteitssingel 50<br>P.O. Box 616<br>6200 MD Maastricht<br><br>RIKILT – Institute of Food Safety<br>P.O. Box 203<br>6700 AE Wageningen<br><br>Division of Human Nutrition<br>Wageningen University<br>P.O. Box 8129<br>6700EV Wageningen

TABLE OF CONTENTS

1. INTRODUCTION AND RATIONALE 11
2. OBJECTIVES 15
3. STUDY DESIGN 16
4. STUDY POPULATION 20
4.1 Population (base) 20
4.2 Inclusion criteria 20
4.3 Exclusion criteria 20
4.4 Sample size calculation 22
5. TREATMENT OF SUBJECTS 23
5.1 Investigational product/treatment 23
6. METHODS 24
6.1 Study parameters/endpoints 24
6.1.1 Main study parameter/endpoint 24
6.1.2 Secondary study parameters/endpoints 24
6.1.3 Other study parameters 24
6.2 Study Procedures 25
6.3 Randomisation and Blinding 27
6.4 Study Parameter Methodology 28
6.4.1 Physiological Measurements 28
6.4.2 Anthropometrical Measurements 30
6.4.3 Biochemical Measurements 30
6.5 Withdrawal of individual subjects 32
6.6 Replacement of individual subjects after withdrawal 32
6.7 Follow-up of subjects withdrawn from treatment 32
6.8 Premature termination of the study 32
7. SAFETY REPORTING 33
7.1 Section 10 WMO event 33
7.2 Adverse and serious adverse events 33
7.3 Follow-up of adverse events 34
8. STATISTICAL ANALYSIS 35
9. ETHICAL CONSIDERATIONS 36
9.1 Regulation statement 36
9.2 Recruitment and consent 36
9.3 Benefits and risks assessment, group relatedness 37
9.4 Compensation for injury 37
9.5 Incentives 37
10. ADMINISTRATIVE ASPECTS AND PUBLICATION 38
10.1 Handling and storage of data and documents 38
10.2 Amendments 38
10.3 End of study report 38
10.4 Public disclosure and publication policy 39
11. REFERENCES 41
Appendix I: List of Flavonoid-Rich Foods and Diet Restrictions 45
Appendix II: Supplement Safety Reports 46

LIST OF ABBREVIATIONS AND RELEVANT DEFINITIONS

**ABR**	**ABR form, General Assessment and Registration form, is the application form that is required for submission to the accredited Ethics Committee (In Dutch, ABR = Algemene Beoordeling en Registratie)**
**ADMA**	**Asymmetric Dimethylarginine**
**AE**	**Adverse Event**
**AIx**	**Augmentation Index**
**ALAT**	**Alanine Transaminase**
**ALP**	**Alkaline Phosphatase**
**AR**	**Adverse Reaction**
**ASAT**	**Aspartate Aminotransferase**
**CA**	**Competent Authority**
**CCMO**	**Central Committee on Research Involving Human Subjects; in Dutch: Centrale Commissie Mensgebonden Onderzoek**
**CHD**	**Coronary Heart Disease**
**CRP**	**C-Reactive Protein**
**CV**	**Curriculum Vitae**
**CVD**	**Cardiovascular Disease**
**DBP**	**Diastolic Blood Pressure**
**DSMB**	**Data Safety Monitoring Board**
**EID**	**Endothelium-Independent Dilation**
**EPI**	**Epicatechin**
**EU**	**European Union**
**EudraCT**	**European drug regulatory affairs Clinical Trials**
**FMD**	**Flow-Mediated Dilation**
**GCP**	**Good Clinical Practice**
**Hb A1C**	**Glycated Hemoglobin**
**HFC**	**High Flavan-3-ol Cocoa**
**IB**	**Investigator's Brochure**
**IC**	**Informed Consent**
**IL-6**	**Interleukin-6**
**IL-8**	**Interleukin-8**
**IMP**	**Investigational Medicinal Product**
**IMPD**	**Investigational Medicinal Product Dossier**
**MCP**	**Monocyte Chemoattractant Protein**
**METC**	**Medical research ethics committee (MREC); in Dutch: medisch ethische toetsing commissie (METC)**
**NO**	**Nitric Oxide**
**PBMC**	**Peripheral Blood Mononuclear Cell**
**PLA**	**Placebo**
**PWA**	**Pulse Wave Analysis**

**PWV**	**Pulse Wave Velocity**
**QUER**	**Quercetin-3-glucoside**
**RBC**	**Red Blood Cell count**
**(S)AE**	**(Serious) Adverse Event**
**SEVR**	**Subendocardial Viability Ratio**
**sICAM-1**	**Soluble Intercellular Adhesion Molecule-1**
**sVCAM-1**	**Soluble Vascular Cell Adhesion Molecule-1**
**SBP**	**Systolic Blood Pressure**
**SPC**	**Summary of Product Characteristics (in Dutch: officiële productinfomatie IB1-tekst)**
**Sponsor**	**The sponsor is the party that commissions the organisation or performance of the research, for example a pharmaceutical company, academic hospital, scientific organisation or investigator. A party that provides funding for a study but does not commission it is not regarded as the sponsor, but referred to as a subsidising party.**
**SUSAR**	**Suspected Unexpected Serious Adverse Reaction**
**sVCAM-1**	**Soluble Vascular Cell Adhesion Molecule-1**
**TNF-α**	**Tumor Necrosis Factor-α**
**vWF**	**Von Willebrand Factor**
**WBC**	**White Blood Cell Count**
**Wbp**	**Personal Data Protection Act (in Dutch: Wet Bescherming Persoonsgevens)**
**WMO**	**Medical Research Involving Human Subjects Act (in Dutch: Wet Medisch-wetenschappelijk Onderzoek met Mensen**
**ɣ-GT**	**Gamma-Glutamyl Transpeptidase**

SUMMARY

Rationale: Epidemiological evidence supports the hypothesis of a protective effect of flavonoids on cardiovascular disease (CVD). To date, most intervention studies have investigated the effects of flavonoid containing-foods (such as tea and cocoa) on cardiovascular health while studies using pure flavonoids have frequently used high doses. As such, few studies have been conducted investigating the effects of dietary doses of pure flavonoids on markers of cardiovascular health in a large population over a prolonged period of time.

Objective: To determine the acute and chronic effects of pure flavonoid supplementation on markers of vascular function in (pre)hypertensive subjects.

Study design: The study will be conducted as a three-armed, randomized, placebo-controlled, double-blind, cross-over trial.

Study population: 40 apparently healthy men and women between the ages of 30 and 80, with untreated (pre)hypertension will be enrolled on the study. (Pre)hypertension will be defined as a systolic blood pressure between 125 and 160 mmHg.

Intervention: The study will be separated into two parts. Part 1 will investigate the effects of pure flavonoid supplements while Part 2 will investigate the effects of a flavanol-rich coca drink.

For Part 1 of the study, participants will first complete a 1-week run-in period during which they will be advised to avoid consumption of foods high in flavonoids. Upon completion of the run-in period, participants will be sequentially randomized to the following three intervention arms for four weeks;

1) 160mg per day quercetin-3-glucoside in capsule form;
2) 100mg per day epicatechin in capsule form;
3) Placebo in capsule form

A washout period of 4 weeks will be included between the interventions, during which participants will be advised to maintain the dietary advice of restricted flavonoid consumption. Measurements of vascular and endothelial function will be taken before and after each intervention arm. In addition, measurements will be taken before, and 2 hours after consumption of the supplements, on the final study day of each intervention.

Four weeks after completion of the last intervention arm, participants will be asked to take part in Part 2 of the study. Part 2 will be optional and participation will not be obligatory for

the participants. Those who opt to take part in Part 2 will be asked to return to the university on two different mornings – separated by two weeks. Measurements of vascular and endothelial function will be taken before and two hours after consumption of either a high or a low-flavanol cocoa beverage. These measurements will be used to compare the effects of pure epicatechin and epicatechin in cocoa on FMD and will also serve as positive control for FMD.

Main study parameters/endpoints: The primary study parameter is the percentage change in Flow-Mediated Dilation (FMD) before and after the intervention period. Additional study parameters include (biochemical) markers of vascular and endothelial function as well as systolic and diastolic blood pressure.

Nature and extent of the burden and risks associated with participation, benefit and group relatedness: The risks of participating in the study are negligible, while the burden is low. For all supplements used, a safety report is provided (see Appendix II). The dosage used for the supplements is consistent with 3 times the P90 of the habitual intake of the Dutch population. As such, the dosage used is in line with what can be achieved through a high-flavonoid diet.

All participants will be asked to visit the university for an initial information session as well as a screening session. During the study, participants will attend six measurement days – separated by 4 weeks. Blood pressure and FMD measurements will be taken at all visits. Blood samples will be taken at all visits, except during the information session – this can be considered the greatest burden for participants. FMD can be considered a small burden due to minor discomfort during the measurement as a result of venous occlusion for 5 minutes and the necessity to remain still during the reading. Six 24-hour urine samples will be collected and 24-hour ambulatory blood pressure will be measured which could be considered an inconvenience to participants.

1. INTRODUCTION AND RATIONALE

Cardiovascular disease (CVD) has been shown to be the leading cause of death and is responsible for approximately 30% of all deaths worldwide [1, 2]. Within the EU alone, CVD costs the economy 192 billion Euros per year and, worldwide, accounts for approximately 17 million deaths per year (the highest of all chronic diseases) with this number predicted to rise to 25 million by 2020 [3, 4].

Nutrition is a key risk factor, as well as prevention strategy, for CVD. Epidemiological studies have demonstrated an inverse association between diets high in fruits and vegetables and CVD [5-7]. In the Zutphen study, for example, it was shown that tea, cocoa and wine were strongly inversely related to CVD [8-11]. Meta-analyses of prospective cohort studies confirmed these inverse associations for tea and wine and showed that these drinks were associated with a 10-30% reduction in CVD risk [12-14].
One ecological observation of interest, includes the low prevalence of CVD within the Kuna Indian population - native to the San Blas Islands off the coast of Panama. Commonly, they consume more than 5 cups of flavanol-rich cocoa per day, which is thought to be one of the underlying explanations for the reduced risk of hypertension and CVD [15]. This is supported by epidemiological studies suggesting that the consumption of dark chocolate can lower blood pressure and is associated with a lower incidence of CVD [8, 16]. The high flavonoid content of these drinks and plant foods has been used as the root of the hypothesis linking their consumption with reduced CVD.

Flavonoids (a subclass of polyphenols) are structurally-related secondary metabolites that are ubiquitous in plant foods. Over 4000 flavonoids have been identified in plant foods and this list is still growing [17]. Flavonoids can be sub-divided (based on their chemical structure) into 6 separate categories; flavan-3-ols, flavonols, flavones, isoflavones, flavanones and anthocyanidins.

The first epidemiological study which studied the relationship between flavonols and CHD mortality found that a high intake compared to a low intake of flavonols was associated with a 68% reduction in risk [9]. A meta-analysis of 7 prospective studies concluded that a high compared to a low intake of flavonols was associated with a more modest, but still significant, 20% lower risk of total CHD [18]. Another meta-analysis of 6 prospective cohort studies showed that stroke incidence was 20% lower in individuals with a high compared to a low flavonol intake [19]. Finally, the intake of monomeric flavan-3-ols (catechin, epicatechin, gallocatechin, epigallocatechin, epicatechin gallate and epigallocatechin gallate) has been

shown to be associated with a 51% lower incidence of CHD [20]. In contrast, however, two other prospective studies failed to establish a decreased association with fatal CHD [21, 22].

It is thought that flavonoids elicit cardio-protective effects through a variety of mechanisms including improving endothelial and vascular function as well as anti-inflammatory and anti-thrombotic responses [23-27]. Due to the large variety of flavonoids within and between food types, the majority of these studies fail to establish a link between pure flavonoids and CVD risk factors. In addition, many studies, especially those investigating the effects of chocolate on CVD risk factors, fail to use an adequate control substance thereby preventing subject-blinding [28-30]. The large number of different flavonoids present in plants and plant foods makes it important to establish which flavonoids are most responsible for the observed cardio-protective effect.

In the western world, flavan-3-ols are often the most commonly consumed flavonoid sub-group. On average, 108mg of flavan-3-ols are consumed per day as compared to 6.4mg and 1.52mg for isoflavones and flavones respectively [31]. Tea (C*amelia sinensis)* and cocoa *(Theobroma cacao)* have been identified as key contributors to dietary flavan-3-ol intake [32]. As previously mentioned, inverse associations have been found between cocoa intake and CVD – suggesting a potential contribution of flavan-3-ols to cardiovascular risk reduction [8, 33]. Similar associations have been found for tea consumption and CHD and CVD risk [33, 34]. Flavan-3-ols exist in many different forms and, as such, it is important to determine which forms are likely to contribute to the beneficial role in cardiovascular health before firm conclusions can be drawn.

In contrast to larger flavan-3-ol molecules, flavan-3-ol monomers (including epicatechin and catechin) are thought to affect cardiovascular health due to their higher absorption and bioavailability [35]. Procyanidins (oligomeric flavan-3-ols) with more than two favan-3-ol units are not absorbed through the intestinal wall and will reach the colon where they are degraded into smaller molecules by gut microflora [36]. Flavan-3-ol monomers found in dark chocolate (such as epicatechin) can be absorbed in the small intestine resulting in peak plasma concentration within two hours of intake [25]. Plasma concentrations of epicatechin have also been found to be significantly higher than other flavan-3-ol monomers such as catechin following cocoa consumption [37]. Considering the decrease in CVD risk factors following dark chocolate or cocoa consumption, the high prevalence of flavan-3-ols found in cocoa and the sharp increase in plasma epicatechin concentrations (as opposed to catechin and procyanidins) it is reasonable to suggest that epicatechin is one of the primary flavonoids responsible for the observed cardio-protective effects.

Acute intake of pure epicatechin has been shown to increase nitric oxide (a potent vasodilator) and decreases endothelin-1 (vasoconstrictor) [38]. In addition, increases in flow-mediated dilation as a result of a high-flavan-3-ol cocoa drink coincided with increases in circulating epicatechin and its metabolite epicatechin-7-*O*-glucuronide [39].

Besides flavan-3-ols, tea is also the major dietary source of flavonols in tea drinking countries - and quercetin is by far the most important dietary flavonol [40]. Flavonols are only present in plant foods as glycosides, i.e. a flavonol conjugated with one or more sugar units. The unconjugated form is called the aglycone. Quercetin-3-glucoside has an increased bioavailability - explained largely by the glucose moiety which is thought to increase absorption in the small intestine [41]. Supplementation of pure quercetin aglycone reduced blood pressure, although no studies have yet been done to investigate the effects of quercetin-3-glucoside on vascular function [42, 43].

The limited number of trials investigating the effects of pure flavonoid intake on markers of cardiovascular health have been performed with high flavonoid doses, in a limited number of subjects and for a short duration [38, 42]. Thus, further studies are required to ascertain the effects of chronic consumption of pure flavonoids (in upper dietary doses) on a variety of vascular function markers in a substantial number of subjects.

We plan to conduct an intervention study using epicatechin and quercetin-3-glucoside - at an intake of 3 times the 90th percentile of habitual intake. In addition, the acute effects of a high flavan-3-ol cocoa drink will be assessed. The high flavan-3-ol cocoa drink will serve purely as a positively control as the effects of high flavan-3-ol cocoa on vascular function and blood pressure have already been demonstrated in a number of intervention studies [27, 44-46].

To investigate the effects, epicatechin and quercetin-3-glucoside supplements will be provided to (pre)hypertensive subjects in the form of a randomized, placebo-controlled, double-blind, cross-over trial. The chronic effects of flavonoid supplementation will be determined on a variety of cardiovascular health markers, including markers of vascular and endothelial function.

If specific flavonoids can be identified as playing a beneficial role in cardiovascular health, this could allow the development of natural flavonoid supplements. Alternatively, specially developed functional foods could be developed by conserving the flavonoid presence during processing.

2. OBJECTIVES

Primary Objective:

- To determine the acute and chronic effects of pure epicatechin and quercetin-3-glucoside on vascular and endothelial function – as measured by Flow-Mediated Dilation.

Secondary Objectives:

- To determine the acute and chronic effects of pure epicatechin and quercetin-3-glucoside on additional functional markers of vascular function – as measured by blood pressure, pulse wave velocity, pulse wave analysis and vasomotion.
- To determine the acute and chronic effects of pure epicatechin and quercetin-3-glucoside on biomarkers of vascular function (e.g. plasma nitric oxide levels, endothelin-1 and ADMA).
- To determine the acute and chronic effects of pure epicatechin and quercetin-3-glucoside on biomarkers of endothelial function (e.g. sVCAM-1, sICAM-1 and sE-selectin).
- To determine the acute and chronic effects of pure epicatechin and quercetin-3-glucoside on biomarkers of inflammation (e.g. Il-6, IL-8 and TNF-α).
- To determine the effects of pure epicatechin and quercetin-3-glucoside on markers of renal function by estimation of glomerular filtration rate (eGFR).
- To determine the effects of epicatechin and quercetin-3-glucoside intake on gene expression using peripheral blood mononuclear cells (PBMCs).
- To compare the effects of pure epicatechin and epicatechin in cocoa on FMD.

3. STUDY DESIGN

Part 1 (Supplement Study):

The study will be undertaken as a three-armed, placebo-controlled, double-blind, cross-over trial. At the start of the study, participants will be asked to follow a 1-week run-in period during which they will be given dietary advice to avoid the consumption of flavonoid-rich foods. Upon completion of the run-in period, participants will follow three 4-week intervention periods with each intervention arm separated by a 4-week washout period. Four weeks after completion of the last intervention arm, participants will be asked to return to the university on two different mornings – separated by two weeks. Measurements of vascular and endothelial function will be taken before and two hours after consumption of either a high or a low-flavanol cocoa beverage. These measurements will serve purely as a positive control for Flow-Mediated Dilation.

Those interested in taking part in the study will first be asked to attend an information session, during which the study will be explained and preliminary blood pressure measurements will be taken. Those deemed eligible based on the blood pressure measurements will be invited to a screening session. During the screening session blood samples will be taken, blood pressure, height, weight and waist circumference will be measured and participants will be asked to complete a short medical history and lifestyle questionnaire. Blood samples taken during the screening will be used to analyse biochemical parameters mentioned in the inclusion criteria (see chapter 4.2). Based on the results of the screening session, those deemed eligible will be asked to take part in the study – which will start with a one-week run-in period. During the one week run-in period, participants will be given dietary guidelines to restrict the consumption of certain flavonoid-rich foods (see Appendix I). This dietary advice will be maintained throughout the study period.

Forty pre-hypertensive subjects will be sequentially randomised to one of the three intervention arms depending on their allocated treatment sequence (see table 5.1). For the three treatments, subjects will be asked to ingest either 160mg of quercetin-3-glucoside (QUER), 100mg of epicatechin (EPI) or placebo capsules (PLA) per day. Supplements will be provided in the form of capsules which will be ingested twice daily – at lunch and in the evening with dinner and with a glass of water.

Baseline measurements will be taken on the first morning of each intervention arm, following an overnight fast (T_0). Hereafter, participants will be asked to consume two capsules per day for four weeks. After the four-week intervention period, follow-up measurements will be taken

at the same time of day as the baseline measurements, again following an overnight fast (T_1). This will be done in order to determine the chronic effects of pure flavonoid consumption. Following these follow-up measurements, participants will be asked to consume two final capsules (i.e. the daily dose). Two hours after consumption of these capsules, additional measurements will be taken in order to determine the acute effects of flavonoid supplementation (T_A). Changes in values between T_0 and T_1 will allow the analysis of the chronic effects of the intervention as compared to placebo over the 4-week period while T_0 and T_A will allow analysis of the acute effects during a 2-hour timeframe. For an overview of the timeline of the intervention period and the measurements to be taken, please refer to figure 3.1 and table 3.1.

Participants will be asked to collect 24-hour urine samples during the 24-hour period preceding the baseline and follow-up measurements (6 collections in total). This will be done in order to measure compliance. In addition, 24-hour ambulatory blood pressure measurements will be taken during the 24-hour period preceding baseline and follow-up measurements.
Halfway between each intervention period (i.e. two weeks after the start of each intervention/washout period) participants will be asked to revisit the university ($T_{½}$). During these visits, participants will be weighed and a blood sample will be taken. These measurements will be taken at the same time of day as the baseline and follow-up measurements and will last no more than 15 minutes.

Two days prior to the follow-up measurements, participants will be asked to come to the university in order to collect a standardised meal as well as materials for urine collection and 24-hour ambulatory blood pressure monitors. The standardised meal will be consumed the evening prior to the baseline and follow-up measurements.

<u>Part 2 (Cocoa Study):</u>
Four weeks after completion of the last intervention arm, participants will be asked to take part in Part 2 of the study. Part 2 will be optional and participation will not be obligatory for the participants. Those who opt to take part in Part 2 will be asked to return to the university on two different mornings – separated by two weeks. Measurements of vascular and endothelial function will be taken before and two hours after consumption of either a high or a low-flavanol cocoa beverage. These measurements will be used to compare the effects of pure epicatechin and epicatechin in cocoa on FMD and will also serve as positive control for FMD. For these measurements, blood samples will also be taken for analysis of nitric oxide and plasma flavonoid concentrations.

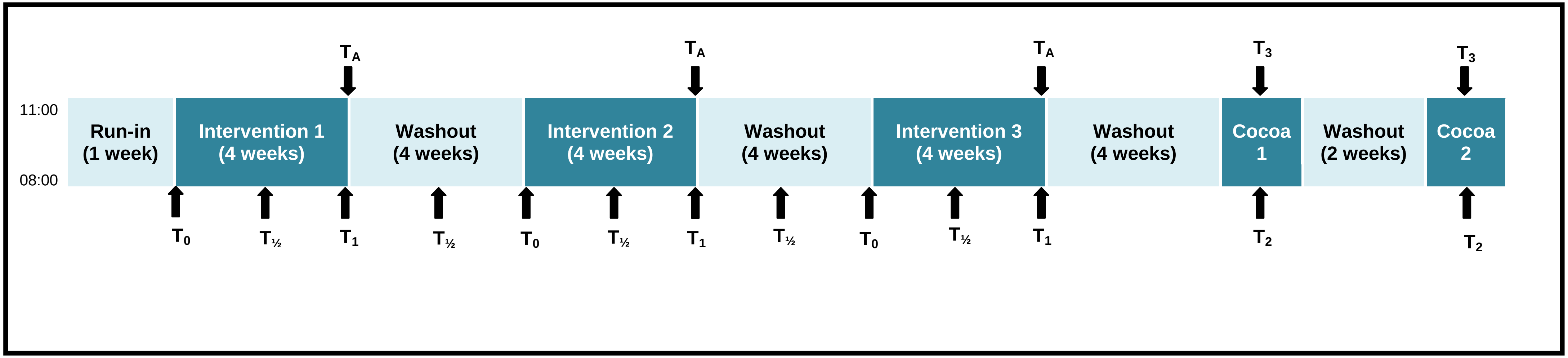

Figure 3.1. Schematic overview of intervention plan and points of measurements. Time times used here are only used as an example.

Table 3.1. Measurements to be taken during the FLAVO-studie.

Measurement	Specimen	Screening	T_0	T_A	$T_{½}$	T_1	T_2 & T_3
Anthropometrics							
Height	N/A	X					
Weight	N/A	X	X	X	X	X	X
Macrovascular Function Markers							
Blood Pressure (systolic, diastolic and central)	N/A	X	X	X		X	X
24-hour Ambulatory Blood Pressure	N/A		X			X	
Heart Rate	N/A	X	X			X	
Flow Mediated Dilation	N/A		X	X		X	X
Endothelium-Independent Dilation	N/A		X	X			X
Microvascular vasomotion	N/A		X	X		X	X
Pulse Wave Analysis (PWA)	N/A		X			X	
Pulse Wave Velocity (PWV)	N/A		X			X	
Biomarkers of Vascular Function							
Nitric Oxide (NOx)	Plasma		X	X		X	X
Endothelin-1	Plasma		X	X		X	
Asymmetric Dimethylarginine (ADMA)	Plasma		X	X		X	
Biomarkers of Endothelial							
sVCAM-1 & sICAM-1	Plasma		X			X	
sE-selectin	Plasma		X			X	
Soluble Thrombomodulin	Plasma		X			X	
Von Willebrand Factor	Plasma		X			X	
Biomarkers of Inflammation							
C-Reactive Protein	Plasma		X			X	
Serum Amyloid A	Plasma		X			X	
Interleukin-6	Plasma		X			X	
Interleukin-8	Plasma		X			X	
Tumor Necrosis Factor-α	Plasma		X			X	
Monocyte Chemoattractant Protein -1	Plasma		X			X	
Biomarkers of Compliance							
Plasma Concentrations Epicatechin and Quercetin	Plasma		X	X	X	X	X
Excreted Epicatechin and Quercetin	Urine		X			X	
Markers of Renal Function							
Estimated Glomerular Filtration Rate (Creatinine)	Plasma		X			X	
Other							
Glucose & Insulin	Plasma		X			X	
Hb A1C and blood cell count	Plasma		X			X	
Lipids and lipoproteins	Plasma		X			X	
Peripheral Blood Mononuclear Cell Gene Expression	N/A		X			X	
Blood safety parameters (ALAT, ASAT, γ-GT, ALP)	Plasma	X	X	X	X	X	X
Protein and albumin excretion	Urine	X	X			X	

4. STUDY POPULATION

4.1 Population (base)

A total of 40 apparently healthy, untreated (pre)hypertensive men and women will be sourced from the Wageningen population and surroundings. Selection of subjects will be based on the exclusion and inclusion criteria detailed below.

4.2 Inclusion criteria

Both men and women will be accepted onto the study providing they are between the ages of 30 and 80 and have a systolic blood pressure between 125 and 160 mmHg and Body Mass Index (BMI) score between 20 and 40 kg/m^2. All subjects must meet the inclusion and not meet the exclusion criteria (see tables 4.1 and 4.2 respectively).

Table 4.1. Inclusion criteria for short-term flavonoid study.

Inclusion Criteria
• Systolic Blood Pressure between 125 and 160 mmHg<br>• Age between 30 and 80 years<br>• BMI > 20 and ≤ 40<br>• Apparently healthy:<br>- No reported current or previous metabolic diseases<br>- No history of cardiovascular diseases<br>- No history of renal, liver or thyroid diseases<br>- No history of gastrointestinal diseases<br>- No diabetes mellitus<br>- Fasting laboratory parameters within normal range: complete blood count (RBC, WBC, PLT, HB, HT), renal function (serum creatinine, ureum), liver function (ALAT, ASAT, ɣ-GT), and serum glucose.

4.3 Exclusion criteria

Before the start of the study:

Subjects who do not meet the inclusion criteria or who have one or more of the exclusion criteria before the start of the study will be deemed ineligible and will be excluded from taking part. See table 4.2 for an overview of the exclusion criteria.

During the intervention:

Subjects who experience a serious adverse event, such as hospitalisation, will be removed from the study. For a definition of a serious adverse event, see chapter 7.2. In addition, subjects whose systolic blood pressure rises above 160 mmHg during the intervention period will be informed of their blood pressure reading and the study physician will contact the subject's General Practitioner (GP). These subjects will be removed from the study if the GP deems it necessary. If the GP decides to prescribe blood pressure-lowering medication then these subjects will also be removed from the study.

Table 4.2. Exclusion criteria for short-term flavonoid study.

Exclusion Criteria
• Body mass index > 40 and ≤20
• Secondary hypertension
• Weight loss or weight gain of 5 kg or more during the last 2 months
• Usage of non-steroidal anti-inflammatory drugs (acetylsalicylic acid, ibuprofen, naproxen) and not able or willing to stop taking them from at least 4 weeks prior to the study
• Usage of cholesterol-lowering medication
• Daily usage of corticosteroids
• Medical treatment that may affect blood pressure and not able (or willing) to stop
• Taking nutritional supplements and unwilling to discontinue
• Lactating, pregnant or intend to become pregnant during study
• Reported dietary habits: medically prescribed diet, slimming diet;
• Reported average alcohol consumption > 2 glasses/d (men) or >1 glass/d (women)
• Problems with consuming the supplements or following the study guidelines
• Unwilling to undergo home or office blood pressure measurements
• Recent blood donation i.e. 1 month (male subjects) or 2 months (female subjects) prior to the study and planned donation during the study period
• Reported intense sporting activities > 10 h/w
• Not agreeing to be informed about unexpected and medically relevant personal test-results, or not agreeing that their general practitioner will be informed about these results
• Participation in another biomedical trial less than 2 months before the start of the study or at the same time
• No signed informed consent form
• Clinical disorders that could interfere with the intervention
• Unable to comply with the study procedure (e.g. holidays, urine collection, blood sampling)
• Smokers
• Difficulty imaging brachial artery by ultrasonography

4.4 Sample size calculation

With FMD as the primary endpoint, a calculation was carried out to determine optimal sample size for the trial. To date, no studies have been conducted investigating the effects of pure flavonoid supplements in doses proposed for this study. For this reason, the sample size calculation will be based on results from studies conducted using cocoa containing an equivalent amount of epicatechin. Based on these previous studies, a standard deviation of the treatment response of 2.7 is assumed for FMD [23, 25].

Studies investigating the acute effects of cocoa consumption report an increase in FMD of approximately 1.5% – which will be used as the expected effect size for the acute effects of the cocoa intervention. Assuming a power of 0.80 and α=0.025, and based on a crossover intervention design with multiple testing, 25 subjects would be required to identify a change in FMD of 1.5%. Assuming a dropout rate of approximately 10%, the optimal sample size to investigate the acute effects of flavanol-rich cocoa is 30.

Studies investigating the chronic effects of cocoa consumption report an increase in FMD of approximately 1.3% – which will be used as the expected effect size for the chronic effects of the intervention. Assuming a power of 0.80 and α=0.025, and based on a crossover intervention design with multiple testing, 35 subjects would be required to identify a change in FMD of 1.3%. Assuming a dropout rate of approximately 10%, the optimal sample size to investigate the chronic effects is 40.

The following formula was used to calculate sample size:

$n = 7.9 \times (SD_{response} / D)^2$	*n= sample size*<br>$SD_{response}$ *= expected standard deviation of the response to treatment*<br>*D = expected effect size due to treatment*

Figure 4.1. Equation used to calculate sample size.

5. TREATMENT OF SUBJECTS

5.1 Investigational product/treatment

The three intervention arms (QUER, EPI, and PLA) will each use different investigational products. During the QUER intervention, participants will be required to take supplements of quercetin-3-glucoside (160mg/d) in the form of capsules. Epicatechin supplements (100mg/d), also in the form of capsules, will be provided during the EPI intervention arm. These dosages are based on three times the 90th percentile of epicatechin and quercetin intake [20, 40]. Finally, capsules containing microcrystal cellulose and 1% colloidal siliciumdioxide will be used as placebo capsules. The flavonoid capsules will also contain microcrystal cellulose as an excipient. To prevent differentiation in appearance, the placebo capsules will be produced by the same company where the flavonoid capsules will be made (*Het Gelre Apotheek, Apeldoorn*). All supplements will be encapsulated in non-transparent capsules and will be matched for appearance and size (capsule size “0”). During the 4-week intervention period, two capsules will be consumed per day - one at the lunch and the second in the evening before dinner. Participants will be asked to consume the capsules with a meal and a glass of water.

Two hours after the follow-up measurements, participants will be asked to consume two additional capsules with a glass of water. This will be done to determine the acute effects of flavonoid supplementation on markers of cardiovascular health.

Four weeks after completion of the last intervention arm, participants will be asked to return to the university on two different mornings – separated by two weeks. Measurements of vascular and endothelial function will be taken before and two hours after consumption of either a high or a low-flavanol cocoa beverage. Consumption of the low or high-flavanol cocoa will be randomly assigned. The high-flavanol cocoa beverage will be matched such that it provides the same amount of epicatechin as the daily dose of epicatechin during the intervention (i.e. 100mg).

During the study, participants will be provided with a list of high-flavonoid containing foods (see Appendix I). They will be asked to avoid over-consumption of such food products during the intervention period. Specifically, participants will be asked to consume no more than 1 cup of tea or glass of red wine per day and to avoid the consumption of chocolate, apples and onion soup.

6. METHODS

6.1 Study parameters/endpoints

6.1.1 Main study parameter/endpoint

The main study parameter is the percentage change in FMD before and after the intervention period.

6.1.2 Secondary study parameters/endpoints

Secondary study parameters include;

- Changes in blood pressure as measured by systolic, diastolic, central and 24-hour blood pressure.
- Changes in macrovascular function as measured by PWA and PWV as well as microvascular function as measured by vasomotion.
- Changes in biomarkers of vascular function as assessed by concentrations of Nitric Oxide, endothelin-1 and ADMA etc.
- Changes in biomarkers of endothelial function as assessed by measurement of plasma levels sVCAM-1, sICAM-1, sE-selectin etc.
- Changes in biomarkers of inflammation as assessed by measurement of plasma levels of CRP, IL-6, IL-8, TNF-α etc.
- Changes in PBMC gene expression as a measurement of gene expression of inflammation.
- Changes in renal function as estimated from glomerular filtration rate (GFR) using serum creatinine.
- Changes in markers that affect vascular function as measured by plasma concentrations of lipoproteins.
- Changes in insulin resistance as measured using HOMA-IR through plasma insulin and glucose levels.

6.1.3 Other study parameters

Anthropometric measurements including height and weight will be measured. Height and weight measurements will be used to determine individual BMI scores.

6.2 Study Procedures

Information Session and Screening:

After expressing interest in taking part in the study, all potential participants will be invited to an initial information session. During this information session, subjects who fulfil the general requirements of the study will be asked to undergo a standard SBP and DBP measurement. Subjects with a blood pressure lower than 125 mmHg at the information session will be excluded from the study.

All potential participants who meet the blood pressure criteria at the information session will be given a short medical history and lifestyle questionnaire as well as informed consent form which they will be allowed to fill in at home and will be invited back for a screening session at the university. Prior to the screening session, participants will be asked to collect 24-hour urine samples. This will be done to determine the level of protein and albumin in the urine. Bottles for urine collection will be provided to all participants.

During the screening itself, height, weight and blood pressure will be measured and blood samples will be taken during the screening session. If a blood pressure score above 160mmHg is measured during the screening session then subjects will be advised to contact their General Practitioner and will not be eligible for participation. Blood samples taken during the screening session will be used to analyse biochemical parameters of general health including kidney and liver function – as stated in the inclusion criteria. Finally, subjects will be tested for feasibility of ultrasound image acquisition on the brachial artery.

Run-in:

Based on the medical history and lifestyle questionnaire as well as the screening measurements, 40 eligible participants will be selected and enrolled onto the study. Prior to the initiation of the first intervention arm, each subject will complete a one-week run-in period. During this run-in period, participants will be asked to restrict their consumption of flavonoid-rich foods (see Appendix I). This dietary advice will be maintained throughout the study period.

Part 1(Supplement Study):

During the 24-hour period preceding the first measurement day, participants will be asked to collect 24-hour urine samples and 24-hour ambulatory blood pressure will be measured. Instructions and bottles for urine collection as well as blood pressure monitors will be provided. Participants will return the blood pressure monitors when they visit the university for the measurement day.

Baseline measurements of all parameters will be taken on the morning of the first study day, following an overnight fast (T_0).

Upon completion of the first study day, participants will be asked to consume two supplements per day for the following 4 weeks before returning for follow-up assessments (T_1). Follow-up assessments will be carried out at the same time of day as the baseline measurements, following an overnight fast and will include the same measurements as the baseline measurements. This means that weight, FMD, PWA, PWV, blood pressure and microcirculation vasomotion will be measured and blood samples will be taken for all participants. After follow-up measurements have been taken, subjects will be asked to consume two capsules (i.e. the daily dosage) of the allocated supplement. Two hours after consumption, additional measurements of FMD, blood pressure, vasomotion and certain biochemical parameters will be taken (T_A).

In preparation for the follow-up assessments, participants will be asked to collect a 24-hour urine sample and 24-hour blood pressure will be measured. Bottles for urine collection and blood pressure monitors will be provided.

Following each intervention period, participants will undergo a washout period during which they will be asked to refrain from consuming high flavonoid-containing foods. During the whole study period, participants will be asked to keep a short diary of certain food records and illnesses. Although participants will be asked to refrain from consuming flavonoid-rich foods, if it so happens that a participant does consume a flavonoid-rich food, they will be asked to record this in the diary. In addition, they will also be asked to monitor and report symptoms of illnesses. As an extra measurement of compliance, participants will also be asked to report days in which they forget to consume the supplement. In order to aid compliance, two weeks' worth of capsules will be provided in a blister pack (Memory Pac, Unit Dose System).

Intermediate Measurements:

Every two weeks participants will be asked to visit the university in order to record their weight, take a blood sample and (if necessary) receive additional supplements for the following two weeks. These intermediate measurements will primarily be planned to keep participants actively involved in the study as well as to measure compliance and monitor health status (liver and renal function as well as whole blood cell count). Participants will be asked to bring all leftover supplements with them, thereby giving an indication of compliance. In addition, a blood sample will be taken and will be analysed to determine the level of plasma flavonoid metabolites.

Part 2 (Cocoa Study):

Four weeks after completion of the last intervention arm, participants will be asked to take part in Part 2 of the study. Part 2 will be optional and participation will not be obligatory for the participants. Those who opt to take part in Part 2 will be asked to return to the university on two different mornings – separated by two weeks. Measurements of vascular and endothelial function will be taken before and two hours after consumption of either a high or a low-flavanol cocoa beverage. These measurements will be used to compare the effects of pure epicatechin and epicatechin in cocoa on FMD and will also serve as positive control for FMD. Blood samples will also be taken for analysis of nitric oxide and plasma flavonoid concentrations .

6.3 Randomisation and Blinding

Randomisation:

Subjects will be sequentially randomly allocated to the various intervention arms based on their allocated treatment sequence (see table 5.1). Due to the fact that there are three intervention arms, six possible treatment sequences exist . Allocation to treatment sequence will be performed by an independent investigator employed at Wageningen University, who is not involved in the study. To maintain confidentiality, this investigator will be provided with an identification number for each participant and will not come into contact with the participants. At the start of each intervention period, another researcher will provide the participants with the materials needed for their allocated intervention. This researcher will not be involved in the collection of anthropometrical, physiological or biochemical data.

Table 5.1. Possible treatment sequences for the FLAVO-study.

Treatment sequence			
1	Placebo	Epicatechin	Quercetin
2	Placebo	Quercetin	Epicatechin
3	Epicatechin	Placebo	Quercetin
4	Epicatechin	Quercetin	Placebo
5	Quercetin	Epicatechin	Placebo
6	Quercetin	Placebo	Epicatechin

Blinding:

Researchers and medical staff will remain blinded towards the intervention. The company providing the capsules for the intervention (Het Gelre Apotheek, Apeldoorn) will be asked to deliver the capsules with a specific code for each treatment. The company will be asked to keep a record of the coding and will provide this code upon completion of the study. In case of serious adverse events, a subject's GP may request unblinding of the treatment.
Upon completion of the study, subjects will be asked to report the sequence of intervention groups they thought they had been allocated to. In addition, all subjects will be informed of the correct sequence of interventions they were allocated to – this will be conducted by the same researcher who provided the subjects with their materials during the intervention.

6.4 Study Parameter Methodology

6.4.1 Physiological Measurements

FMD and EID: To reduce variation, Flow-Mediated Dilation (FMD) and Endothelium-Independent Dilation (EID) measurements will always be conducted by the same researcher. FMD will be measured at time points T_0, T_A, T_1, T_2 and T_3. EID will be measured at T_0, T_A and T_3.
FMD will be measured as the percentage change in artery diameter in response to 5 minutes of venous occlusion. After baseline images have been obtained during a 3-minute rest period, a blood pressure cuff (placed distal to the antecubital fossa) will be inflated to 200mmHg for 5 minutes – thereby causing arterial occlusion. Brachial artery images will be recorded by means of ultra-sonography for 5 minutes after deflation.
EID will be measured as the percentage change in artery diameter in response to sublingual nitroglycerin administration (400µg). EID will be measured in order to distinguish between the endothelium-dependent and endothelium-independent effects of the intervention. Subjects will be warned of the side-effects of nitroglycerin administration prior to the start of the study. Side effects may include headaches and dizziness. Subjects will be advised to rest for 15 minutes after administration and to avoid intense exercise immediately after the measurement.

The percentage change in FMD/EID will be calculated as follows:

$$\% \text{ FMD/EID} = \frac{\text{(maximum diameter – baseline diameter)}}{\text{baseline diameter}} \times 100$$

Blood Pressure: Systolic and diastolic blood pressure (SBP and DBP respectively) will be measured at the information session, screening session and at time points T_0, T_A and T_1 during the study.
For each measurement, four automatic blood pressure readings will be taken at 2-minute intervals (DINAMAP® PRO 100). The first reading will be discarded and an average of the final three will be calculated. Central blood pressure (CBP) will only be measured at time points T_0 and T_1 - using the SphygmoCor CPV System (AtCor Medical, Australia). Ambulatory 24-hour blood pressure (Spacelabs Healthcare) will be measured during the 24-hour period before the start and end of each intervention arm.

Pulse Wave Analysis (PWA): Pulse Wave Analyses will only be conducted at time points T_0 and T_1. This means that a total of six Pulse Wave Analyses will be conducted per subject. For each analysis, three measurements will be taken and the average will be used to calculate the final values.
A variety of variables will be measured using the SphygmoCor CPV System (AtCor Medical, Australia). PWA allows a non-invasive assessment of the cardiovascular system by applanation of a tonometer on the radial pulse. From this measurement, CBP, augmentation index (AIx), subendocardial viability ratio (SEVR) and ejection duration will be automatically calculated.

Pulse Wave Velocity (PWV): Using the same apparatus (SphygmoCor CPV System), PWV will be measured at the same time points as PWA. The tonometer will sequentially be placed on the right carotid and femoral arteries for 15 seconds at a time – from which the PWV will be calculated. For each artery, three measurements will be taken. The average of the three measurements will be used for the final PWV calculation. Measurements will be taken in a temperature-controlled room following at least 10 minutes of rest. The distance between carotid and femoral artery will be measured using an infantometer.

Microvascular Vasomotion: Vasomotion will be conducted at time points T_0, T_A, T_1, T_2 and T_3. Microvascular vasomotion provides an assessment of blood perfusion as a result of rhythmic oscillations in vascular tone in small vessels of the circulatory system (capillaries, arterioles and venules) and will be measured using a Periflux 5001 Laser Doppler System (Perimed AB, Sweden). In brief, a laser Doppler probe is attached to the back of the non-dominant hand which releases a beam of laser light. Moving particles, such as blood cells, alter the wavelength of the returning signal allowing for the calculation of the number and velocity of circulating blood cells.

6.4.2 Anthropometrical Measurements

Body Weight: Subjects will be weighed using a digital balance scale, accurate to 0.1kg. All participants will be asked to wear minimal clothing, remove shoes and empty their pockets for weighing. Subjects will be weighed at the screening and at time points T_0, T_A, $T_{½}$, T_1 and T_2 during the study.

Height: Height will be measured during the screening session using a configured wall-mounted stadiometer accurate to 0.5cm. All participants will be measured during the screening; wearing no shoes, with their back against the wall and with their head in the Frankfurt plane.

6.4.3 Biochemical Measurements

Fasting blood samples will be collected at all time points. This means that a total of 14 samples will be taken during the study. At time points T_0 and T_1, 50ml of blood will be collected. At time points T_A, $T_{1/2}$, T_2 and T_3, 20ml of blood will be collected.
All blood samples will be collected by trained lab technicians. Based on these blood samples, various plasma biomarkers will be analysed. All participants will be informed about the possible risk of bruising as a result of venepuncture.

Markers of Endothelial and Vascular Function: Endothelin-1 and ADMA will be analysed by Ultra-Performance Liquid Chromatography Tandem Mass Spectropometry (UPLC-MS/MS). Von Willebrand Factor (vWF) will be determined in citrated plasma by means of ELISA. All other biomarkers will be analysed by an electrochemiluminescence detection system using multiarray technology (Meso Scale Discovery). These analyses will be conducted at the central lab (i.e. Department of Internal Medicine, Maastricht University), with the exception of nitric oxide (NO) which will be measured at the RIKILT Institute of Food Safety. This will be done based on the estimation of NO formed by chemiluminescence after the release of nitric oxide from nitroso compounds and nitrosated and nitrosylated species

Renal Function: Glomerular Filtration Rate (GFR) will be estimated using the Modification of Diet in Renal Disease (MDRD) equation. This will be done based on values obtained for serum creatinine.
The MDRD equation is as follows:

$$\text{GFR (ml/min/1.73m}^2\text{)} = 186.3 \times (\text{Serum creatine in mg/dl})^{-1.154} \times \text{age}^{-0.203} \times 1.212 \text{ (if patient is black)} \times 0.742 \text{ (if female)}$$

Serum Lipoproteins, Glucose and Screening Samples: From the collected plasma samples, total cholesterol and high-density lipoprotein (HDL) as well as glucose will be measured at an external lab – the Stichting Huisartsen Lab Oost (SHO) in Velp, the Netherlands. Samples during screening will also be analysed at SHO for RBC, WBC, PLT, HB, HT (complete blood count), Hb A1C as well as ALAT, ASAT and ɣ-GT (for liver function) and serum creatinine and ureum (for renal function).

Gene Expression: RNA will be isolated from peripheral blood mononuclear cells (PBMCs) to determine the level of gene expression of markers of inflammation and endothelial function.

Insulin Resistance and Sensitivity: Fasting plasma insulin and glucose levels will be used to estimate insulin resistance and sensitivity using the Homeostasis Model Assessment (HOMA-IR). Plasma insulin concentrations will be determined by radioimmunoassay at Wageningen University.

Plasma Flavonoids: Plasma flavonoid concentrations will be determined by means of HPLC-electrochemical detection at the RIKILT – Institute for Food Safety.

Measurement of Compliance: Due to the short half-life of flavonoids in blood, both urine and plasma concentrations of flavonoid metabolites will be measured to assess compliance. Plasma and 24-hour urine samples will be collected before the start of the study and on the last day of each intervention period. Both samples will be analysed by High-Pressure Liquid Chromatography. Additional plasma samples will be taken every two weeks as well as before, and 2 hours after, consumption of the final supplement on the last day of each intervention period.

6.5 Withdrawal of individual subjects

Subjects can leave the study at any time for any reason if they wish to do so without any consequences. The investigator can decide to withdraw a subject from the study for urgent medical reasons.

6.6 Replacement of individual subjects after withdrawal

If subjects withdraw prior to the start of the first intervention arm, they may be replaced if an appropriate replacement can be enrolled in time. Subjects who withdraw after the start of the first intervention period will not be replaced.

6.7 Follow-up of subjects withdrawn from treatment

Subjects who wish to withdraw from the study will be asked about their reason for withdrawal and whether they would still be willing to attend a follow-up assessment despite stopping the intervention. These subjects will also be asked if they would be willing to remain on the university's list of potential study participants for future studies.

6.8 Premature termination of the study

The standardized operating procedure of the Division of Human Nutrition concerning premature termination of the study will be used.

7. SAFETY REPORTING

7.1 Section 10 WMO event

In accordance to section 10, subsection 1, of the WMO, the investigator will inform the subjects and the reviewing accredited METC if anything occurs, on the basis of which it appears that the disadvantages of participation may be significantly greater than was foreseen in the research proposal. The study will be suspended pending further review by the accredited METC, except insofar as suspension would jeopardise the subjects' health. The investigator will take care that all subjects are kept informed.

7.2 Adverse and serious adverse events

Prior to the start of the study, all participants will be screened based on a number of health factors. As such, subjects with a blood pressure greater than 160mmHg will be excluded from the study. If participation in the study results in an increase in blood pressure above 160 mmHg then the study physician (Dr. Marco Mensink) will contact the participant's general practitioner (GP). If the GP deems it necessary to withdraw the participant from the study, then they will be free to do so. Alternatively, if the GP prescribes blood pressure-lowering medication, the participant will also be removed from the study.

If abnormalities are reported based on blood analyses during the screening or after completion of the study, then participants will be informed.

Adverse events are defined as any undesirable experience occurring to a subject during the study, whether or not considered related to [the investigational product / the experimental treatment]. All adverse events reported spontaneously by the subject or observed by the investigator or his staff will be recorded. These adverse events will then be reported to the study physician who will determine if they are a result of the intervention or if they could have detrimental effects to the results of the study.

A serious adverse event (SAE) is defined as any untoward medical occurrence or effect that at any dose:

- results in death;
- is life threatening (at the time of the event);
- requires hospitalisation or prolongation of existing inpatients' hospitalisation;
- results in persistent or significant disability or incapacity;
- is a congenital anomaly or birth defect;

- is a new event of the trial likely to affect the safety of the subjects, such as an unexpected outcome of an adverse reaction, lack of efficacy of an IMP used for the treatment of a life threatening disease, major safety finding from a newly completed animal study, etc.

All SAEs will be reported through the web portal *ToetsingOnline* to the accredited METC that approved the protocol, within 15 days after the sponsor has first knowledge of the serious adverse reactions.
SAEs that result in death or are life threatening should be reported expeditiously. The expedited reporting will occur not later than 7 days after the responsible investigator has first knowledge of the adverse reaction. This is for a preliminary report with another 8 days for completion of the report.

7.3 Follow-up of adverse events

All adverse events will be followed until they have abated, or until a stable situation has been reached. Depending on the event, follow up may require additional tests or medical procedures as indicated, and/or referral to the general physician or a medical specialist.

8. STATISTICAL ANALYSIS

Statistical analyses will be conducted based on the intention-to-treat principle. An additional per-protocol analysis will be conducted, however, the intention-to-treat analysis will remain leading. Researchers involved in the statistical analysis will remain blinded to treatment allocation. For statistical significance, a two-tailed p-value of 0.05 will be set.

All data collected during the study will be quantitative. Continuous variables will be expressed as means ± standard deviation while categorical variables will be expressed in frequencies. Data will be tested for normality by visual inspection of Q-Q plots and histograms.

Basic descriptive analyses (including age, gender, BMI and blood pressure) will be carried out based on subject characteristics obtained during the screening and baseline measurements.

A repeated measures analysis of variance (ANOVA) will be conducted to compare differences in intraindividual responses to the treatments. Changes from T_0 to T_1 will be used to determine the chronic effect of the intervention. Changes from T_1 to T_A will be used to determine the acute effects of the intervention. Additional paired t-tests will be carried out to compare baseline values (T_0) of outcome measures.

For data that are not normally distributed, a nonparametric Friedman's rank test will be conducted. All statistical analyses will be carried out using the SPSS 19.0 or SAS 9.2 software packages.

9. ETHICAL CONSIDERATIONS

9.1 Regulation statement

This study will be conducted according to the Declaration of Helsinki – Ethical Principles for Medical Research Involving Human Subjects (updated October, 2008) and in accordance with the Medical Research Involving Human Subjects Act (WMO) and local regulations.

9.2 Recruitment and consent

Eligible participants will be recruited from the area of Wageningen and the nearby surroundings. Advertisements will be released 2 months prior to the start of the study, in which the general scope and criteria for the study will be mentioned. These advertisements will be released digitally (via email) , by posters and by mail. In addition, participants from a recent similar study (the KaNa-trial) will be asked upon completion of the study, whether they would be interested in taking part in this study. Participants who have completed other nutritional intervention studies at Wageningen University will also be approached.

Subjects who express an interest in taking part will be sent an information brochure explaining the aim and design of the study as well as the inclusion/exclusion criteria required in order to take part. Subjects who are still interested in taking part in the study after having read the information brochure will be provided with contact details in order to arrange an appointment for an information session. Those who do not contact the research team within 2 weeks of receiving the information brochure will be contacted by telephone to see whether they are still interested in taking part in the study.

During the information session, potential participants will be given an initial informed consent form for the information session and screening. Standard SBP and DBP measurements will be taken for all participants who attend the information session. After the information session, potential participants will be given a short medical history and lifestyle questionnaire which they can complete at home. Subjects who meet the criteria based on the health and lifestyle questionnaire, BP measurements and who return a signed informed consent form will be invited to a screening session at the university. This screening session will take place no earlier than 4 weeks before the start of the study. Upon completion of the screening session, those who meet the inclusion criteria and are still interested in taking part will be invited to take part in the study.

9.3 Benefits and risks assessment, group relatedness

The risks for participation in this study are low. For all supplements used, a safety report has been obtained (see Appendix II). A potential burden for the subjects may be the restricted diet (i.e. chocolate, tea, red wine, apples and onions). In addition, subjects will be required to attend 6 assessment sessions (including FMD and BP measurements) following an overnight fast, which could be considered as an additional burden.

9.4 Compensation for injury

The sponsor/investigator has a liability insurance which is in accordance with article 7, subsection 6 of the WMO.

The sponsor (also) has an insurance which is in accordance with the legal requirements in the Netherlands (Article 7 WMO and the Measure regarding Compulsory Insurance for Clinical Research in Humans of 23th June 2003). This insurance provides cover for damage to research subjects through injury or death caused by the study.

1. € 450.000,-- (i.e. four hundred and fifty thousand Euro) for death or injury for each subject who participates in the Research;
2. € 3.500.000,-- (i.e. three million five hundred thousand Euro) for death or injury for all subjects who participate in the Research;
3. € 5.000.000,-- (i.e. five million Euro) for the total damage incurred by the organisation for all damage disclosed by scientific research for the Sponsor as ‘verrichter’ in the meaning of said Act in each year of insurance coverage.

The insurance applies to the damage that becomes apparent during the study or within 4 years after the end of the study.

9.5 Incentives

Subjects who complete the main study (part 1) will receive €250. Subjects who fail to complete the study will be compensated based on the amount of (assessment) days completed. Subjects who complete the additional cocoa study (part 2) will receive an additional €50. The amount of compensation provided will be calculated based on the measurements taken during each assessment day according to pre-determined rates specified by the Division of Human Nutrition at Wageningen University. These rates are based on the degree of burden and length of duration of the measurement. In addition,

participants will receive compensation for travel costs based on the estimated distance travelled.

10. ADMINISTRATIVE ASPECTS AND PUBLICATION

10.1 Handling and storage of data and documents

Each participant will be provided with a randomly-assigned individual identification code. To ensure confidentiality, only the researchers involved in the study will have access to the identity codes. During statistical analyses, all values will be coded by the identification code, thereby ensuring anonymity.

All data will be stored digitally on the internal network of the Division of Human Nutrition at Wageningen University. This internal network will remain password-protected and the password will only be made available to the investigators, research assistants and potential graduate students involved in the study after completion of a confidentiality agreement.

Hard copies (e.g. completed questionnaires and reports of physiological measurements) will be kept for a duration of 24 months after completion of the study, after which all copies will be destroyed. Blood samples will be stored in a locked freezer at Wageningen University and will be coded by the identification code. This study will be registered at the *College Bescherming Persoonsgegevens* (College Protection Personal Information).

10.2 Amendments

Amendments are changes made to the research after a favourable opinion by the accredited METC has been given. All amendments will be notified to the METC that gave a favourable opinion.

10.3 End of study report

The investigator will notify the accredited METC of the end of the study within a period of 8 weeks. The end of the study is defined as the last patient’s last visit. In case the study is ended prematurely, the investigator will notify the accredited METC - including the reasons for the premature termination.

Within one year after the end of the study, the investigator/sponsor will submit a final study report with the results of the study, including any publications/abstracts of the study, to the accredited METC.

10.4 Public disclosure and publication policy

The results of this study will be submitted for publication in international scientific journals and will constitute a part of James Dower's final PhD dissertation. In addition, the sponsor is entitled to review and provide comments on manuscripts and/or articles prior to publication. The sponsor may delay publication for up to three months after analysing the research results if it is applying for a patent or for other important reasons. Participants can receive a summary of the results with averages of the group after the end of the study. Finally, the trial itself will be registered at ClinicalTrials.gov and will be allocated a specific trial registration number.

11. REFERENCES

1. WHO. *Cardiovascular Diseases (CVDs): Fact Sheet N^{0} 317*. 2011 20-07-2011]; Available from: http://www.who.int/mediacentre/factsheets/fs317/en/index.html.
2. Allender, S., Scarborough, P., Peto, V., Rayner, M., Leal, J., Luengo-Fernandez, R., Gray, A., *European Cardiovascular Disease Statistics 2008*, 2008: Brussels.
3. Rayner, M., S. Allender, and P. Scarborough, *Cardiovascular disease in Europe.* European Journal of Cardiovascular Prevention and Rehabilitation, 2009. **16**(SUPPL. 2): p. S43-S47.
4. Yach, D., et al., *The global burden of chronic diseases: Overcoming impediments to prevention and control.* Journal of the American Medical Association, 2004. **291**(21): p. 2616-2622.
5. Dauchet, L., et al., *Fruit and vegetable consumption and risk of coronary heart disease: A meta-analysis of cohort studies.* Journal of Nutrition, 2006. **136**(10): p. 2588-2593.
6. He, F.J., et al., *Increased consumption of fruit and vegetables is related to a reduced risk of coronary heart disease: Meta-analysis of cohort studies.* Journal of Human Hypertension, 2007. **21**(9): p. 717-728.
7. Joshipura, K.J., et al., *Fruit and vegetable intake in relation to risk of ischemic stroke.* Journal of the American Medical Association, 1999. **282**(13): p. 1233-1239.
8. Buijsse, B., et al., *Cocoa intake, blood pressure, and cardiovascular mortality: The Zutphen Elderly Study.* Archives of Internal Medicine, 2006. **166**(4): p. 411-417.
9. Hertog, M.G.L., et al., *Dietary antioxidant flavonoids and risk of coronary heart disease: The Zutphen Elderly Study.* Lancet, 1993. **342**(8878): p. 1007-1011.
10. Keli, S.O., et al., *Dietary flavonoids, antioxidant vitamins, and incidence of stroke: The Zutphen study.* Archives of Internal Medicine, 1996. **156**(6): p. 637-642.
11. Streppel, M.T., et al., *Long-term wine consumption is related to cardiovascular mortality and life expectancy independently of moderate alcohol intake: The Zutphen Study.* Journal of Epidemiology and Community Health, 2009. **63**(7): p. 534-540.
12. Arab, L., W. Liu, and D. Elashoff, *Green and black tea consumption and risk of stroke: A meta-analysis.* Stroke, 2009. **40**(5): p. 1786-1792.
13. Di Castelnuovo, A., et al., *Meta-Analysis of Wine and Beer Consumption in Relation to Vascular Risk.* Circulation, 2002. **105**(24): p. 2836-2844.
14. Peters, U., C. Poole, and L. Arab, *Does tea affect cardiovascular disease? A meta-analysis.* American Journal of Epidemiology, 2001. **154**(6): p. 495-503.
15. Hollenberg, N.K., N.D.L. Fisher, and M.L. McCullough, *Flavanols, the Kuna, cocoa consumption, and nitric oxide.* Journal of the American Society of Hypertension, 2009. **3**(2): p. 105-112.
16. Buijsse, B., et al., *Chocolate consumption in relation to blood pressure and risk of cardiovascular disease in German adults.* European Heart Journal, 2010. **31**(13): p. 1616-1623.
17. Hollman, P.C.H. and M.B. Katan, *Dietary flavonoids: Intake, health effects and bioavailability.* Food and Chemical Toxicology, 1999. **37**(9-10): p. 937-942.
18. Huxley, R.R. and H.A.W. Neil, *The relation between dietary flavonol intake and coronary heart disease mortality: A meta-analysis of prospective cohort studies.* European Journal of Clinical Nutrition, 2003. **57**(8): p. 904-908.
19. Hollman, P.C.H., A. Geelen, and D. Kromhout, *Dietary flavonol intake may lower stroke risk in men and women.* Journal of Nutrition, 2010. **140**(3): p. 600-604.
20. Arts, I.C.W., et al., *Catechin intake might explain the inverse relation between tea consumption and ischemic heart disease: The Zutphen Elderly Study.* American Journal of Clinical Nutrition, 2001. **74**(2): p. 227-232.
21. Mink, P.J., et al., *Flavonoid intake and cardiovascular disease mortality: A prospective study in postmenopausal women.* American Journal of Clinical Nutrition, 2007. **85**(3): p. 895-909.
22. Mursu, J., et al., *Flavonoid intake and the risk of ischaemic stroke and CVD mortality in middle-aged Finnish men: The Kuopio Ischaemic Heart Disease Risk Factor Study.* British Journal of Nutrition, 2008. **100**(4): p. 890-895.

23. Davison, K., et al., *Effect of cocoa flavanols and exercise on cardiometabolic risk factors in overweight and obese subjects.* International Journal of Obesity, 2008. **32**(8): p. 1289-1296.
24. Di Giuseppe, R., et al., *Regular consumption of dark chocolate is associated with low serum concentrations of C-reactive protein in a healthy italian population.* Journal of Nutrition, 2008. **138**(10): p. 1939-1945.
25. Engler, M.B., et al., *Flavonoid-rich dark chocolate improves endothelial function and increases plasma epicatechin concentrations in healthy adults.* Journal of the American College of Nutrition, 2004. **23**(3): p. 197-204.
26. Oyama, J.I., et al., *Green tea catechins improve human forearm vascular function and have potent anti-inflammatory and anti-apoptotic effects in smokers.* Internal Medicine, 2010. **49**(23): p. 2553-2559.
27. Wang-Polagruto, J.F., et al., *Chronic consumption of flavanol-rich cocoa improves endothelial function and decreases vascular cell adhesion molecule in hypercholesterolemic postmenopausal women.* Journal of Cardiovascular Pharmacology, 2006. **47**(SUPPL. 2): p. S177-S186.
28. Grassi, D., et al., *Cocoa reduces blood pressure and insulin resistance and improves endothelium-dependent vasodilation in hypertensives.* Hypertension, 2005. **46**(2): p. 398-405.
29. Hermann, F., et al., *Dark chocolate improves endothelial and platelet function.* Heart, 2006. **92**(1): p. 119-120.
30. Taubert, D., et al., *Effects of low habitual cocoa intake on blood pressure and bioactive nitric oxide: A randomized controlled trial.* Journal of the American Medical Association, 2007. **298**(1): p. 49-60.
31. Maras, J.E., et al., *Flavonoid intakes in the Baltimore Longitudinal Study of Aging.* Journal of Food Composition and Analysis.
32. Song, W.O. and O.K. Chun, *Tea is the major source of flavan-3-ol and flavonol in the U.S. diet.* Journal of Nutrition, 2008. **138**(8): p. 1543S-1547S.
33. Kuriyama, S., et al., *Green tea consumption and mortality due to cardiovascular disease, cancer, and all causes in Japan: The Ohsaki study.* Journal of the American Medical Association, 2006. **296**(10): p. 1255-1265.
34. De Koning Gans, J.M., et al., *Tea and coffee consumption and cardiovascular morbidity and mortality.* Arteriosclerosis, Thrombosis, and Vascular Biology, 2010. **30**(8): p. 1665-1671.
35. Manach, C., et al., *Bioavailability and bioefficacy of polyphenols in humans. I. Review of 97 bioavailability studies.* The American journal of clinical nutrition, 2005. **81**(1 Suppl): p. 230S-242S.
36. Deprez, S., et al., *Transport of proanthocyanidin dimer, trimer, and polymer across monolayers of human intestinal epithelial Caco-2 cells.* Antioxidants and Redox Signaling, 2001. **3**(6): p. 957-967.
37. Holt, R.R., et al., *Procyanidin dimer B2 [epicatechin-(4β-8)-epicatechin] in human plasma after the consumption of a flavanol-rich cocoa.* American Journal of Clinical Nutrition, 2002. **76**(4): p. 798-804.
38. Loke, W.M., et al., *Pure dietary flavonoids quercetin and (-)-epicatechin augment nitric oxide products and reduce endothelin-1 acutely in healthy men.* American Journal of Clinical Nutrition, 2008. **88**(4): p. 1018-1025.
39. Schroeter, H., et al., *(-)-Epicatechin mediates beneficial effects of flavanol-rich cocoa on vascular function in humans.* Proceedings of the National Academy of Sciences of the United States of America, 2006. **103**(4): p. 1024-1029.
40. Hertog, M.G.L., et al., *Intake of potentially anticarcinogenic flavonoids and their determinants in adults in The Netherlands.* Nutrition and Cancer, 1993. **20**(1): p. 21-29.
41. Manach, C., et al., *Polyphenols: Food sources and bioavailability.* American Journal of Clinical Nutrition, 2004. **79**(5): p. 727-747.
42. Edwards, R.L., et al., *Quercetin reduces blood pressure in hypertensive subjects.* Journal of Nutrition, 2007. **137**(11): p. 2405-2411.
43. Egert, S., et al., *Quercetin reduces systolic blood pressure and plasma oxidised low-density lipoprotein concentrations in overweight subjects with a high-cardiovascular*

disease risk phenotype: A double-blinded, placebo-controlled cross-over study. British Journal of Nutrition, 2009. **102**(7): p. 1065-1074.
44. Almoosawi, S., et al., *The effect of polyphenol-rich dark chocolate on fasting capillary whole blood glucose, total cholesterol, blood pressure and glucocorticoids in healthy overweight and obese subjects.* British Journal of Nutrition, 2010. **103**(6): p. 842-850.
45. Fisher, N.D.L., et al., *Flavanol-rich cocoa induces nitric-oxide-dependent vasodilation in healthy humans.* Journal of Hypertension, 2003. **21**(12): p. 2281-2286.
46. Heiss, C., et al., *Sustained increase in flow-mediated dilation after daily intake of high-flavanol cocoa drink over 1 week.* Journal of Cardiovascular Pharmacology, 2007. **49**(2): p. 74-80.

Appendix I: List of Flavonoid-Rich Foods and Diet Restrictions

Tijdens de studie wordt u gevraagd om bepaalde voedselproducten die een hoge concentratie aan flavonoïden bevatten te vermijden. Als u veel van deze producten consumeert, zal uw flavonoïdeninname stijgen waardoor het moeilijk wordt om een onderscheid te maken tussen de effecten van de supplementen en de effecten van de flavonoïden uit uw dieet.

Producten die een hoge concentratie aan flavonoïden bevatten zijn ***thee, cacao, chocola, appels, uien*** en ***rode wijn***. Dit zijn de producten waar u rekening mee moet houden tijdens de FLAVO-studie. Graag willen wij u vragen om (zo ver als mogelijk is) deze producten te vermijden. Thee hoeft u niet helemaal te vermijden, hier mag u ***één kop per dag*** van drinken.

Vóór het begin van de studie zult u een dagboek ontvangen waarin u bepaalde gebeurtenissen kunt vermelden worden. Mocht het gebeuren dat u per ongeluk meer dan één kop thee of (bijvoorbeeld) een appel eet, kan dit in het studie dagboek genoteerd worden.

Tijdens de gehele studie geldt het volgende dieetadvies:

1. Niet meer dan één kop **thee** per dag drinken.
2. Geen **appels** (of appelproducten zoals appelsap, appelgebak, appelmoes) eten/drinken.
3. Geen puur/melk **chocola** eten (of cacaoproducten zoals chocoladetaart, koeken, chocolademelk..). Witte chocolade mag wel gegeten worden.
4. Geen **uiensoep** eten. Andere gerechten waarin uien verwerkt worden (zoals hutspot en hachee) kunnen gerust gegeten worden.
5. Niet meer dan 1 glas **rode wijn** per dag drinken. Witte wijn, rosé wijn en port mag wel gedronken worden.

Mocht u twijfelen of vragen hebben, dan kunt u contact met ons opnemen door op werkdagen te bellen naar 0317-481486 of te emailen naar flavo@wur.nl

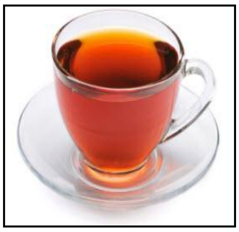
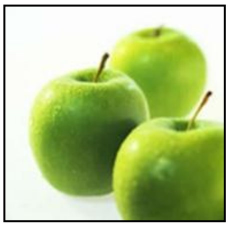
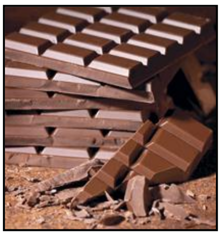
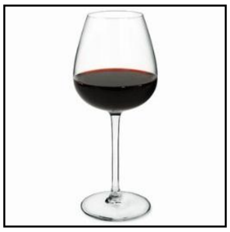
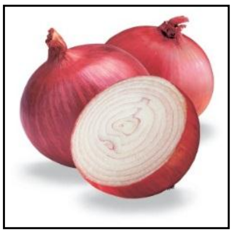

Appendix II: Supplement Safety Reports
